# Preparation of iron oxide mesoporous magnetic microparticles as novel multidrug carriers for synergistic anticancer therapy and deep tumor penetration

**DOI:** 10.1038/s41598-019-46007-z

**Published:** 2019-07-01

**Authors:** Kheireddine El-Boubbou, Rizwan Ali, Hajar Al-Zahrani, Thadeo Trivilegio, Abdullah H. Alanazi, Abdul Latif Khan, Mohamed Boudjelal, Abdulmohsen AlKushi

**Affiliations:** 1 Department of Basic Sciences, College of Science & Health Professions (COSHP), King Saud bin Abdulaziz University for Health Sciences (KSAU-HS), King Abdulaziz Medical City, National Guard Health Affairs, Riyadh, 11481 Saudi Arabia; 2 King Abdullah International Medical Research Center (KAIMRC), King Abdulaziz Medical City, National Guard Health Affairs, Riyadh, 11426 Saudi Arabia; 3 Department of Pathology and Laboratory Medicine, King Abdulaziz Medical City, National Guard Health Affairs, Riyadh, 11426 Saudi Arabia

**Keywords:** Targeted therapies, Drug delivery, Targeted therapies

## Abstract

The preparation of mesoporous iron oxides with controllable physiochemical properties for effective therapeutic drug delivery remains a formidable challenge. Herein, iron oxide mesoporous magnetic microparticles (IO-MMMs) were prepared by a modified reverse hard-templating approach using, for the first time, acid-prepared mesoporous spheres (APMS) as the hard silica template. The obtained mesostructures exhibited remarkably high surface area and large pore volumes (*S*_*BET*_ = 240 m^2^/g and *V*_*pore*_ = 0.55 cm^3^/g), controllable average sizes, generally uniform morphologies, and excellent biocompatibilities, allowing them to achieve optimal drug release in cancer cells and tumor tissues. IO-MMM carriers were able to co-load high amounts of hydrophilic chemotherapeutic drugs (Dox or Daun) and/or hydrophobic hormonal anticancer drugs (Tam), and release them sustainably in a pH-dependent manner, utilizing the fluorescence of Daun to real-time trace the intracellular drug distribution, and employing Daun/Tam to treat cancer by combined chemo/hormonal therapy. Cytotoxicity assays against different types of cancerous cells showed that the combinatory Daun/Tam@IO-MMM formulation significantly reduced the viability of metastatic MCF7 and KAIMRC1 breast as well as HCT8 colorectal cancer cells, with the least potency towards non-cancerous normal primary cells (up to 10-fold). Electron, flow, and live confocal microscopy imaging confirmed that the loaded vehicles were successfully and differentially uptaken by the different tested cells, gradually releasing their payloads, and causing apoptotic cell death. Importantly, compared to free drugs, Daun/Tam@IO-MMMs displayed enhanced drug accumulation in patient breast primary tumor tissues, deeply penetrating into the tumor region and killing the tumor cells inside. The designed carriers described here, thus, constitute a novel promising magnetic mesoporous smart system that entraps different kinds of drugs and release them in a controlled manner for combinatorial chemo/hormonal cancer theranostics. This multifactorial platform may open new avenues in cancer therapy as efficient synergistic antitumor system through overcoming limitations of conventional cancer therapy.

## Introduction

Cancer therapy is a long-standing, formidable and complex process^1^. In most cases, single strategy such as chemotherapy, hormonal therapy, photothermal therapy, or immunotherapy alone is not sufficient to eradicate and eliminate tumors completely. The major challenge remains to establish an advanced carrier platform that can efficiently and selectively destroy cancer cells and penetrate deeply into tumor tissues, releasing their therapeutic payloadsin a controlled manner^2–5^. Consequently, examples of advanced drug delivery systems with chemo/photodynamic, immuno/photothermal, or chemo/immuno therapeutic combinations have been recently investigated with promising potentialsin cancer treatment^6–8^. Despite the many obstacles, however, developing formulations with controlled drug release and synergistic combination therapy can be the optimal strategy for intratumoural drug delivery and enhanced cancer therapeutics.

Of the various drug delivery systems established^9,10^, mesoporous (having pores of 2 to 50 nm) platforms are especially attractive, due to their large surface areas, internal pore volumes, and tunable pore structures allowing large amounts of various drugs to be loaded into their pores at once and subsequently released on demand. Besides, when the construct is further magnetic, the obtained particles will have potential promise in medicine particularly as magneto-responsive drug delivery carriers^11^. In fact, hybrid mesoporous and/or magnetic carriers are gaining immense importance in improving cancer therapies systematically explored in oncology^12,13^. In this context, materials that respond to intracellular pH-driven stimulus have shown to perfectly implement excellent spatial, temporal, and dosage control drug release from different types of carriers with reduced side toxicities, thus, enhancing the efficacy of therapies^14–16^. Consequently, a pH-susceptible iron oxide-based mesoporous material, which is both magnetic and porous, employed as synergistic antitumor platform for cancer therapy is highly desirable. Nonetheless, the impeccable means to synthesize high-quality mesoporous magnetic iron oxide materials with large surface areas and pore volumes remains unambiguously challenging. Thus, preparation of mesoporous iron oxides with controllable physiochemical properties for drug delivery applications is not well established and is explored to less extent. In fact, most mesoporous-based drug delivery systems focus either on mesoporous silica materials or hetero-structured materials, particularly composed of magnetic iron oxide nanoparticles (MNPs) coated with mesoporous silica shells (core/shell), loaded into hollow mesoporous silica nanosphere, or embedded in a mesoporous silica matrix^17–22^. Reports on the utilization of mesoporous iron oxide nano/microstructures for therapeutic drug delivery applications are very rare^23–25^. Importantly, according to our knowledge, mutli-drug delivery using mesoporous magnetic constructs to deliver combination of both hydrophilic and hydrophobic anticancer therapeutic drugs simultaneously has not been reported.

The formation of mesoporous silica, alumino-silicates and related materials are well-known and relies on supramolecular arrays: micellar systems formed by anionic or cationic surfactants^26^ or block copolymers (mainlypolyethylene-based polymers)^27^. However, the synthesis of porous transition metal oxides, particularly iron oxides, has proven to be significantly more difficult and limited^28^, with some important advances seen in recent decades^29^. In general, other than the less used solvothermal or hydrothermal methods which results in very poor porous materials^24,30–32^, mesoporous metal oxide materials are most commonly prepared either *via* “soft-templating method” (templated by surfactants, block copolymers, colloids etc.)^33–36^ or by the “hard-templating method” (i.e. guest materials replicate the “inverse” or “surface” structures of the “host” inorganic materials), where an inverse replica is produced^37–40^. Utilization of hard templates for the preparation of mesoporous metal oxides has some advantages compared with soft templates, especially in its specific topological stability, veracity, predictability, controllability, and crystalline properties^41^. From the various hard templates employed (i.e. alumina membranes, latex spheres, carbon nanotubes, carbon-silica composites etc.), mesoporous silica has already displayed its power to incorporate different guests into their matrices, which led to fabrication of a great variety of nanostructured composites, such as carbon^42^, transition metals^43^, and metal oxides^39,44–47^. Particularly, three-dimensional (3D) and two-dimensional (2D) mesoporous silica (i.e. KIT-6, SBA-15, MSU-H, MCM-41 etc.) with ordered or disordered mesopores and high internal pore surface area have proven to be an ideal host for the syntheses of such materials^48–52^. Moreover, their thermal stability and high silanol density helps to incorporate inorganic metal precursors into their channels by sorption, phase transition, ion exchange, or grafting and, thus, form an inorganic framework by further thermal treatments. For instance, solutions of iron salts (i.e. FeCl_3_or Fe(NO_3_)_3_) are typically used as precursors that are either co-precipitated with the silica source or infiltrated into porous silica^53,54^. The subsequent drying and thermal treatment results in distribution of iron oxide nanoparticles inside the silica matrix, followed by final etching of the silica template to form the desired material. Using this nanocasting templated approach, mesoporous iron oxides(Fe_2_O_3_or Fe_3_O_4_) with ordered and disordered crystalline walls have been prepared *via* impregnation of different iron concentrations into calcined mesostructured silica templates, mainly 3D bicontinuous KIT-6and 2D hexagonally ordered SBA-15^41,48,51,55,56^. However, low surface areas and pore volumes are typically obtained. General challenges in quick crystallization, accompanied with structural collapse during the mesostructure formations, along with the nature of the pore structures within the micellar silica template chosen are considerable issues. Other difficulties lie in the removal of the silica template, where the replicated mesostructure is seldom maintained because the inorganic precursors are inclined to be absorbed on the external surface of templates or the channels are not completely filled, causing the framework formed inside the pores to be lacking in sufficient internal cross-linkages^51,57^.

In this work, we report the preparation of iron oxide mesoporous magnetic microparticles (IO-MMMs) with high surface areas and large internal pore volumes based on a modified reverse hard-templating approach using a novel mesoporous silica as the host. In particular, acid-prepared mesoporous spheres (APMS) are employed for the first time as the hard silica templates. Because of the excellent properties of the APMS template resulting from its rigid framework, 3D interconnected pores, as well as its high chemical and thermal stability, good control over key physiochemical properties of the replicated material is achieved. The as-prepared IO-MMMs exhibit generally spherical shapes, uniform size distributions, high surface areas, and large pore volumes, allowing them to be utilized as efficient multi-drug delivery vehicles. In order to achieve maximal synergistic anticancer effects, co-loading high amounts of one or more therapeutic drug is a necessity to attain simultaneous drug delivery with optimal efficient doses. As a proof-of-concept, anticancer chemo/hormonal therapeutic drugs of various hydrophilicities were loaded into the IO-MMMs and systematically tested against different types of human cancerous cells and patient tumor tissues. Loading and release results show that high loading efficiencies were achieved with drug release rates dramatically improved at low acidic cellular pH, but not at neutral physiological pH. The cytotoxic effects of IO-MMMs and drug@IO-MMMs were then evaluated in different types of breast and colorectal cancer cells as well as normal primary cells. The fluorescence of drugs was monitored by live confocal imaging to demonstrate subcellular internalization and pH-driven drug release of drug@IO-MMMs inside the cells. Moreover, electron microscopy was employed to further assess the uptake of the IO-MMMs-treated cellular samples. Importantly, when tested with primary breast tumor surgery sections derived directly from patient primary lesions, enhanced drug penetration with evident tissue shrinkage was observed, implying that the system is a highly promising combinatory chemo/hormonal therapeutic platform with efficient synergistic antitumor effects. This new platform designed here integrated real-time tracking, pH-driven drug release, and chemo/hormonal therapy, affording optimized efficacy to potentially treat cancer through enhanced passive targeting and combinatorial therapeutic mechanisms. To our knowledge, this is the first report using chemo/hormonal magnetic formulation to systematically study the microparticles’ uptake and co-delivery of their cytotoxic agents to different types and stages of human cancerous cells and tumors.

## Results and Discussion

### Preparation and characterization of IO-MMMs

**Figure 1:**
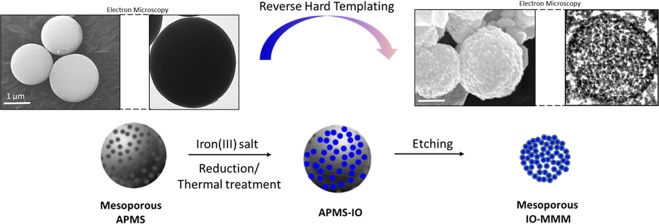
Illustrative representation for the preparation of iron oxide mesoporous magnetic microparticles (IO-MMM). APMS is first prepared as the hard silica template, followed by impregnating the mesopores with iron precursors in the presence of reducing agent, subsequent thermal treatment, and finally removal of the silica template to produce IO-MMM.

Fabrication of IO-MMMs was achieved based on a modified reverse hard-templating nanocasting methodology as outlined in Fig. 1. The first steps involve the production of the hard silica APMS template containing unique 3D pore-connected network and average pore size diameters of ~ 4–6 nm prepared according to our previous work^58,59^. *In situ* reduction of iron(III) precursors during impregnation process, followed by thermal treatment under an inert atmosphere, and finally removal of the silica template generated the desired mesoporous material. In particular, iterative incorporation of APMS silica by aqueous solutions of iron (III) salt (i.e. FeCl_3_) in the presence of a reducing agent, followed by a thermal sintering step produced the continuous iron oxide phase within the pores. Treatment with basic NaOH solution etched away the silica template affording IO-MMM constructs. Repeated impregnation allows maximal incorporation of the aggregated iron precursors filling the continuous mesopore system, which are turned to spherical iron oxide nanoclusters within the silica template during the reduction and heating treatments.

**Figure 2:**
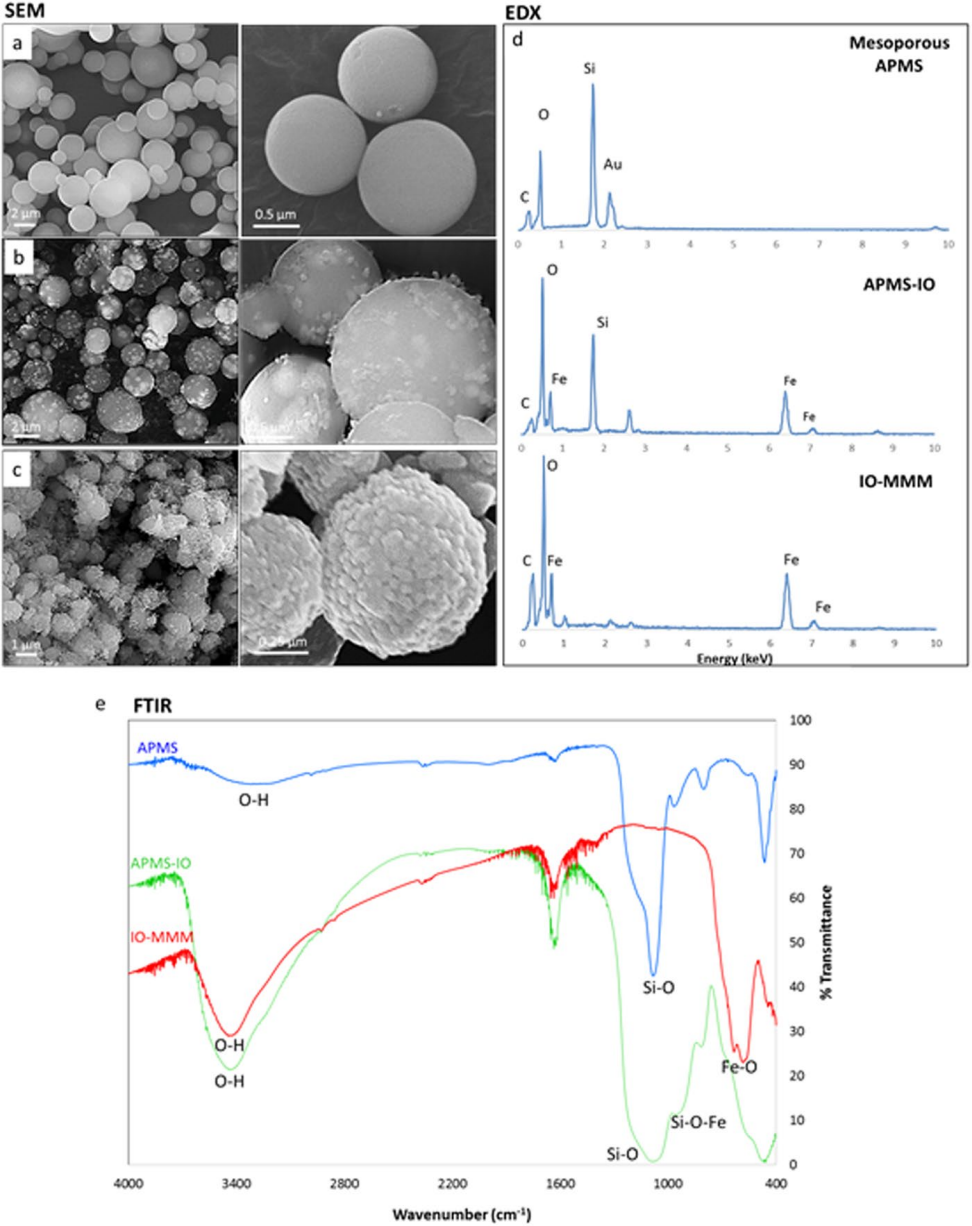
SEM images of (**a**) APMS; (**b**) APMS-IO intermediate; and (**c**) IO-MMM at different magnifications. (**d**) Corresponding EDX patterns of the different samples selected from the various SEM images. (**e**) FRIT spectra for APMS template, APMS-IO intermediate and final IO-MMM. The IR spectra clearly shows the disappearance of the distinctive peaks in the region 960–1100 cm^−1^ corresponding to Si-O (1100 cm^−1^) and Si-O-Fe (960 cm^−1^) stretching vibrations and the appearance of iron oxide Fe-O (~500–600 cm^−1^).

**Figure 3:**
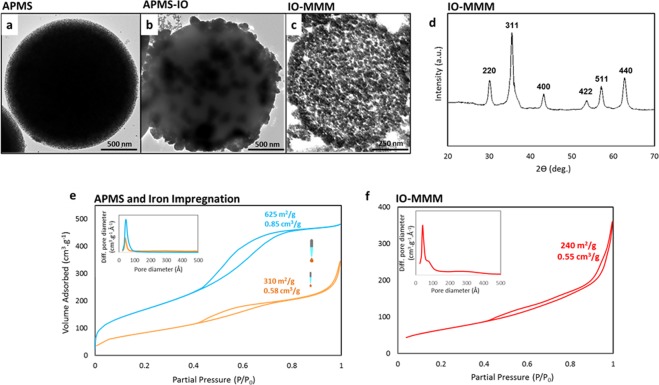
Representative TEM images of (**a**) APMS; (**b**) APMS-IO; and (**c**) IO-MMM. The average size of IO-MMM is 750 nm. (**d**) Wide-angle XRD pattern of IO-MMM. The observed diffraction peaks coincide with the Jade database (JCPDS #039–1346) indicating that the mesoporous iron oxides are maghemite (γ-Fe_2_O_3_). (**e**,**f**) N_2_ adsorption/desorption isotherm curves of (**e**) APMS and its successive impregnation with iron, and (**f**) IO-MMMs with high surface area of 240 m^2^/g and pore-size volume of 0.55 cm^3^/g (inset: respective BJH pore size diameter plots).

The as-prepared IO-MMM mesoconstructs were thoroughly characterized by different spectroscopic techniques including scanning and transmission electron microscopy (SEM and TEM), energy-dispersive X-ray spectroscopy (EDX), nitrogen (N_2_) physisorption porosimetry, X-ray diffraction (XRD), fourier-transform infrared (FTIR), and dynamic light scattering (DLS). SEM images of the APMS silica host, the APMS-IO intermediate, and the templated IO-MMMs at different magnifications are shown in Fig. 2a–c. Typical average APMS sizes of ~1.5 μm with a spherical morphology and practically uniform distribution was observed. While the surface of APMS seem to be clear, smooth, and free of surface cracks, APMS-IO intermediate appeared as IO-dotted architectures, and the final IO-MMM exhibited porous bumpy-like structures. EDX elemental analysis performed on selected SEM areas confirmed the presence of only Fe and O without any other element as impurity in the final replicated mesoporous material (Fig. 2d). EDX measurements on the intermediate samples reveal average atomic Fe/Si ratios of 0.5:1 corresponding to almost equal weight percentages of Fe to Si. This range fits with the relative iron quantities used for impregnation, indicating that iron is distributed quite homogeneously within the samples. FTIR spectroscopy for IO-MMMs clearly showed the distinctive characteristic absorption stretching bands of iron oxide Fe-O (~500–600 cm^−1^) and O-H (~3200–3600 cm^−1^), with the disappearance of peaks in the region 960–1100 cm^−1^ correspondingto Si-O (1100 cm^−1^) and Si-O-Fe (960 cm^−1^) stretching vibrations^60^ (Fig. 2e), confirming the EDX results. TEM images were then recorded to identify the mesostructure of the obtained IO-MMMs (Supplemental Fig. S1). From a TEM image of a single particle, it is clearly indicated that the original mesopore APMS system has been replicated and that the obtained material is composed of interconnected small nanoparticles that possess mesopores (Fig. 3a–c). For APMS-IO intermediate and due to the electronic density contrast, iron oxide nanoclusters (in black) appear to be homogeneously distributed within the mesopores of APMS (in gray) (Fig. 3b). Very few agglomerated iron oxide species were observed independently, suggesting that the impregnation occurred mostly inside the mesopores. In Fig. 3c, the light-contrast areas randomly distributed throughout the microparticle indicate the presence of replicated disordered interconnected porous network and also display the crystallinity of the IO-MMM framework. To further explore the phases and crystalline nature of IO-MMMs, wide-angle powder XRD was performed. XRD data revealed that the produced brown-colored material is γ-Fe_2_O_3_ (maghemite), with observed diffraction patterns in excellent agreement with previously published data for maghemite (JCPDS #039-1346)^39,61,62^ (Fig. 3d). A d-value of 2.96 Å corresponding to the spacing of (220) planes confirmed the presence maghemite^63^. The diffraction peaks are quite low in intensity, signifying that the mesoporous walls are composed of crystalline framework. Most imperious, the porous nature of IO-MMMs was examined by N_2_ adsorption/desorption experiments. As shown in Fig. 3e, successive iron loading (i.e. repetitive iron-impregnation to ensure maximal loading) indicated significant decrease in the surface area and pore volume of APMS-IO compared to the original calcined APMS silica host having Brunauer-Emmett-Teller surface area (*S*_BET_) = 625 m^2^/g and pore volume (*V*_pore_) = 0.85 cm^3^/g, both indications of iron oxide incorporation/coating of the inner pore surfaces of the silica walls. The pore size distribution plots revealed a quitesharp average pore diameters (d_*pore*_) centered at ~5 nm (Fig. 3e inset). Remarkably, N_2_ physisorption isotherm of the final replicated IO-MMM (after thermal treatment and etching) showed that the obtained mesostructured iron oxides exhibit relatively high *S*_*BET*_ of 240 m^2^/g and *V*_*pore*_ of 0.55 cm^3^/g, with broad pore-size distribution having a major *d*_pore_ at ~ 4 nm (Fig. 3f). The isotherm demonstrates a typical Type IV curve characteristic with a well-defined hysteresis loop, indicative of the presence of mesopores and consistent with the recently reported porosities for mesoporous iron oxides^48^. Because of the disorder interconnected nature of channels in APMS, the pore size distribution of the product is somehow broad as a result of random growth and orientation of iron oxide crystals within the APMS template during impregnations and subsequent thermal treatments. The obtained pore-size distributions, however, are in good agreement with the pore diameters anticipated for a replica structure of APMS. According to our knowledge, the as-prepared IO-MMMs obtained here have higher surface areas than any other mesoporous iron oxide material reported in the literature, which is typically between 86 to 187 m^2^/g^48,51,61,63^.

**Figure 4:**
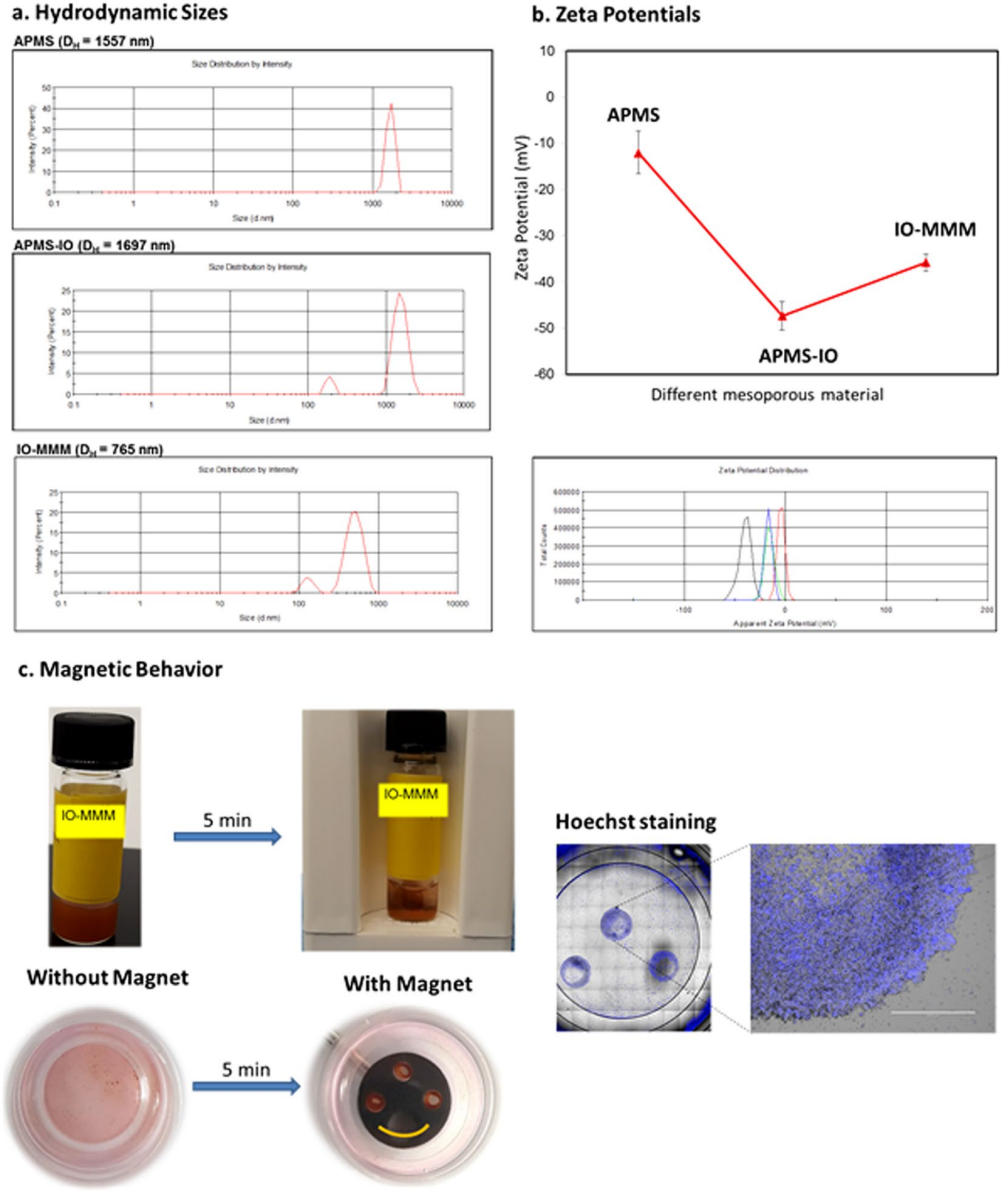
(**a**) Average hydrodynamic sizes and (**b**) average zeta potential measurements of the different constructs (APMS, APMS-IO, and IO-MMM) dispersed in water. The data clearly shows the uniformity of the materials, the incorporation of IO in the pores of silica, and the successful formation of IO-MMMs with narrow size distributions. (**c**) Magnetic behavior of IO-MMMs showing photographs of the separation of IO-MMMs by a magnet from their aqueous dispersions (up) and when treated with cells (down); respective microscopy images of IO-MMM-treated cells stained with nuclei Hoechst in the presence of magnet.

Hydrodynamic particle sizes and zeta potentials were then measured by DLS (Fig. 4a,b). Aqueous dispersion of IO-MMM in water depicted an average hydrodynamic size (D_H_) = 765 nm which is approximately two fold smaller than the average D_H_ (~1500 nm) for APMS. This is expected and agrees well with the N_2_ physisorption results, as replicated mesoporous materials should typically have smaller sizes than their templates. The relatively sharp peaks obtained further confirms the core size obtained by electron microscopy and pinpoints the uniform size distribution and stable dispersity of the as-synthesized IO-MMMs (PDI ~0.5) in water. As expected, the intermediate APMS-IO revealed similar hydrodynamic sizes compared to APMS, suggesting major deposition of iron oxide nanoclusters inside the pores. However, a smaller population at ~190 nm corresponding to 20% of the total intensity was observeddue to iron oxide agglomerates forming independently outside the pores, consistent with TEM results. Notably, zeta potential measurements indicated average zeta potential (ξ) = −12.03 ± 4.62 mV for APMS template, ξ = −45.35 ± 3.07 mV for intermediate APMS-IO, and ξ = −32.5 ± 1.87 mV for IO-MMM, further corroborating successful incorporation of iron oxide and etching of the silica template. All these data combined suggest the assured formation of high surface area and large pore volume IO-MMM mesostructures. Moreover, the high density of hydroxyl groups on IO-MMM surfaces render them water dispersible and suitable for biological assays.

It is worth pinpointing that we initially attempted synthesizing the iron oxide mesoporous constructs by incipient wetting of the APMS template *via* polyacrylic acid-stabilized iron oxide MNPs (PAA-MNPs). Taken into account the strong chelating effect of PAA to the iron oxide surfaces and its previous successful use in the formation of mesoporous iron oxide nanospheres^23^, we thought this will be an effective approach to prepare IO-MMMs. PAA-MNPs were synthesized using our “Ko-precipitation Hydrolytic Basic (KHB)” methodology^64,65^ resulting in uniform, stable, and colloidal ultrasmall MNPs of ~5 nm core diameter. APMS was then impregnated with PAA-MNPs, followed by thermal treatment to facilitate decomposition of PAA polymer and fusion of IONPs. However, it seems that the pore diameters of APMS template are not able to host PAA-MNPs which appear to mainly cluster and aggregate on the APMS surface rather than fill the pores. Nevertheless, TEM and N_2_ physisoprtion isotherms confirmed the presence of some iron oxide mesoporous magnetic nanoclusters (IO-MMNs) with lower surface areas (*S*_BET_ = 88.6 m^2^/g and *V*_pore_ = 0.157 cm^3^/g) and smaller hydrodynamic sizes ~300 nm (Supplemental Fig. S2).

### Magnetic behavior of IO-MMMs

Due to the intrinsic magnetic properties of iron oxide, one of the key advantages of the designed IO-MMMs is their potential use as magnetic imaging probes, magnetically-triggered drug delivery, and clinical hyperthermia^11,19,25^. Magnetic resonance imaging has become the ideal imaging modality to test the biodistibution of magnetically-labeled cells and monitor their fates noninvasively. Furthermore, human tissues are transparent to magnetic field, making the use of external magnet an alternative and powerful approach to overcome limitations of light-enhanced or photo-responsive drug delivery. It was found that IO-MMMs can be easily separated by a magnetic force from their water dispersions within a few minutes, signifying the magnetic characteristics of the mesostructures (Fig. 4c). Furthermore, to test the capabilities of IO-MMMs to label cells, we measured the response of IO-MMM-tagged cells to a static magnetic field. As a proof of principle, KAIMRC1 cells were incubated with IO-MMMs, harvested, and transferred to petri dishes, one of which was located within a static magnetic field (Fig. 4c). Delightfully, it was clear that in the presence of the magnet, cells were concentrated and aligned with respect to the magnetic field lines while in the absence of the permanent magnet, cells were rather disseminated in the petri dish. This demonstrates the possibility of concentrating and spatially locating IO-MMM-tagged cells which is particularly useful for applications such as magnetic cell sorting. This magnetic IO-MMM platform may open new opportunities as promising materials not only for combined chemo/hormonal therapy but also for magnetically-triggered hyperthermia drug delivery applications.

### Drug loading and release

**Figure 5:**
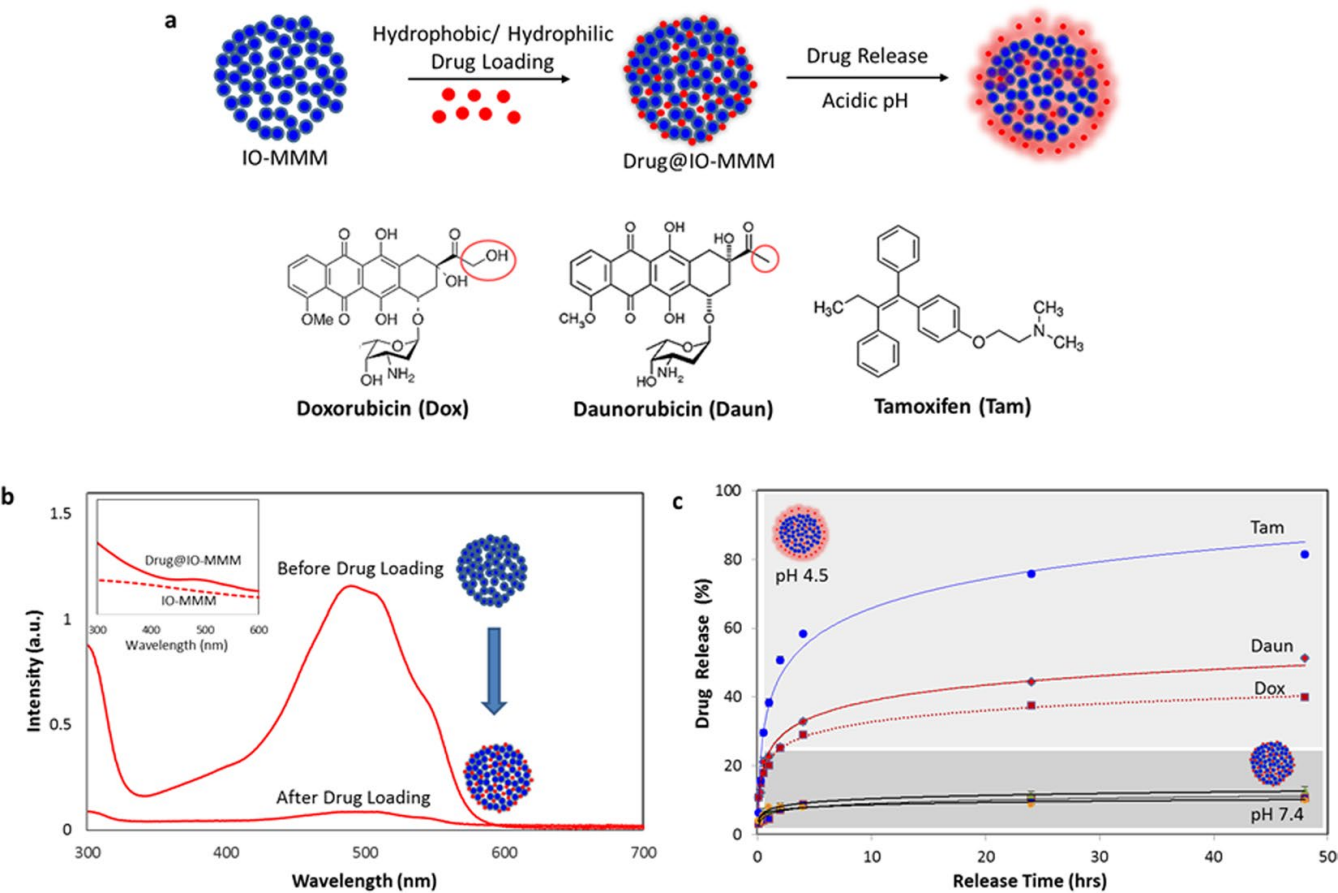
(**a**) Graphical representation of drug loading into IO-MMM and their pH-dependent release. (**b**) Drug loading: Representative UV-vis spectra of drug solution (Dox) before and after loading into IO-MMMs (inset: corresponding UV-vis spectra of aqueous dispersion of Dox@IO-MMMs clearly showing the successful incorporation of Doxinto the IO-MMM mesoconstructs. (**c**) Drug release profiles from drug@IO-MMMs at physiological and slightly acidic pH values. A sustainable controlled release of drugs was observed with Tam being released faster than Dox/Daun due to its hydrophobic nature.

To test the utilization of IO-MMMs as drug carriers, we investigated drug loading of two well-known hydrophilic chemotherapeutic drugs Doxorubicin (Dox) and Daunorubicin (Daun) and a hydrophobic hormonal anticancer drug Tamoxifen (Tam) and studied their pH-dependent release *in vitro* (Fig. 5a). Two different drug formulations (i.e. Dox@IO-MMM and Daun@IO-MMM) along with a combinatory Daun/Tam@IO-MMM formulation were employed. Nowadays in clinical practice, combination chemotherapy is emerging as an important protocol to enhance the therapeutic effects and reduce systemic side toxicities. For instance, Tam is typically used as hormonal therapeutic anticancer drug, in combination with other chemotherapeutic drugs, for the treatment of both early and advanced estrogen receptor-positive breast cancer^66^. Nevertheless, as in the case of many chemotherapeutic drugs, side effects such as increased blood clotting, retinopathy, corneal opacities, and development of endometrial cancer are usually evident with Tam. Moreover, many of the anticancer drugs including Tam are hydrophobic, rising concerns of low solubility, bioavailability, drug toxicity and resistance, thus limiting their tangible clinical outcomes. Ideally for effective therapy, the drug delivery vehicle should carry large amounts of drugs, and then systemically release them to the cancerous cells and diseased tumor tissues in a controlled means. Hence, use of biocompatible systems for efficient delivery of hydrophilic/hydrophobic drugs or even combinatory drugs can provide maximal therapeutic effects with minimal negligent side effects. While co-delivering systems have been explored by precious works^7,31,67,68^, very few studies examined the use of iron oxide-based magnetic particles as drug delivery carriers for Tam^69–71^, and none as a combinatory chemo/hormonal therapeutic vehicle. Herein, simple adsorption of drugs onto IO-MMM mesostructures was chosen, as it has the advantage to preserve both the structure of the constructs and the loaded drugs. Up to 91% of Dox (227 μg Dox/mg of particles), 94% Daun (235 μg Daun/mg of particles), and 83% (165 μg Tam/mg of particles) can be loaded onto IO-MMMs, as evident from absorption spectroscopy (Supplemental Fig. S3a). As expected, the loading efficiencies decreased when higher concentrations of the drug solutions were used (between 75–94% for Dox or Daun and 66–83% for Tam depending on the initial drug concentrations used). Representative UV-vis spectrum of Dox loading onto IO-MMMs to yield Dox@IO-MMMs is shown in Fig. 5b. Furthermore, UV-vis of aqueous dispersion of Dox@IO-MMMs clearly shows the successful loading of drugs into IO-MMMs (Fig. 5b inset). Such high loading efficiencies are much greater than drug incorporation onto nonporous polymer-coated iron oxide MNPs (~80–150 μg of Dox/mg of MNPs) reported in our previous work^72,73^. In fact, the high drug loading amounts of IO-MMM is chiefly attributed to its mesoporous nature with high surface area and pore volume packing the different drug molecules. The broad pore size distribution (dominantly at 4 nm) and the large surface areas of the pores could indeed help load plenty ofanticancer drugs with suitable molecular sizes such as Dox which has a molecular size estimated to be ~1.37 nm^74^. FTIR spectroscopy was further extended to confirm the loading of drugs into the IO-MMM vehicles. Representative FTIR spectra clearly showed that for instance Dox@IO-MMMs has the same characteristic absorption bands as the free Dox with distinctive peaks at ~3400 cm^−1^and ~2900 cm^−1^ due to O-H/N-H stretching and C-H stretching vibrations of the incorporated Dox, respectively (Supplemental Fig. S3b). Moreover, the IR spectrum also showed the disappearance of the bands at 1730 cm^−1^ (corresponding to C-13 carbonyl of Dox) and at ~3520 cm^−1^ (N-H stretching vibrations of the primary amine), indicating the successful incorporation of Dox into IO-MMMs most probably *via* the involvement of -NH_2_, -OH and carbonyl groups of Dox, consistent with previous reports. Daun@IO-MMMs and Daun/Tam@IO-MMMs were also characterized in the same manner and were found to have similar properties. Zeta potential and DLS measurements were also recorded for the three different drug@IO-MMM formulations. Average zeta potential values were found to be ξ = −24.9 ± 1.55 mV for Dox@IO-MMMs, ξ = −26 ± 1.01 mV for Daun@IO-MMMs, and ξ = −7.57 ± 1.65 mV for Daun/Tam@IO-MMMs (Supplemental Fig. S4). Furthermore, size distributions (D_H_ ~830 nm) revealed no significant change in the hydrodynamic sizes, pinpointing the narrow size distribution, homogeneity, and uniformity of the as-synthesized particles after drug loading. In brief, mild shifts in FTIR and lack of new unidentified peaks upon drug loading, along with UV-vis, size, and zeta measurements prove that the drugs are indeed incorporated in the pores of the mesostructures with no significant structural changes occurring to either the drugs or the mesoconstructs upon loading.

With the drug@IO-MMMs in hand, we first investigated their *in vitro* drug release profiles in PBS buffer at two different pH values (Fig. 5c). Release of drugs from the IO-MMM mesostructures was found to be pH-dependent, allowing the drugs to be selectively released within the slightly acidic environments, but not at physiological neutral conditions. The total drugs released from IO-MMM constructs were about 40% Dox, 50% Daun, and 80% Tam at slightly acidic pH of 4.5 after 48 hrs, which is remarkably higher than that at physiological pH of 7.4 (only 7–10% after 48 hrs). In the case of the hydrophobic drug Tam, faster release at low acidic pH was observed compared to a more sustained Dox and Daun release, which might be ascribed to the physical absorption of the drugs inside the nanochannels. The initial burst release in the first hours is typical and is attributed to the rapid diffusion of drug molecules which interact weakly with the outer surfaces or near the pore entrances of the channels. From the release studies, it is evident that at neutral pH the drugs are tightly captured within the large surface areas of IO-MMM pores mainly due to electrostatic, hydrogen bonding, and van der Waals interactions between the drug molecules and particle/pore surfaces containing large number of hydroxyl groups. Due to the protonation of the hydroxyl groups on IO-MMM with the decrease in pH, electrostatic interactions between drugs and IO-MMM will be decreased, causing their release from the IO-MMM surface and mesoporous channels (Supplemental Fig. S5), consistent with previous observations^19,75^. For instance, Dox (pKa = 8.25) is positively charged at neutral pH^76^ and can be adsorbed onto the negatively charged IO-MMMs. Meanwhile, hydrogen bonding between the hydroxyl groups of IO-MMM and the corresponding groups of Dox could be disrupted by adjusting proton amounts at different pH values^30,76–78^. A recent report attributed that Dox release at lower pH is due to the competitive binding of hydronium ions (H_3_O^+^) to the pores of mesoporous iron oxide^25^. Moreover, it has been shown that the coordination between Dox and Fe(III) is more labile at acidic pH, which is also consistent with the observed release at lower pH^79^. All this proves that the drug release is faster at mildly acidic pH values as a consequence of the overall weakened interactions between the drugs and the particle/pore surfaces. This contained drugs at neutral pH with higher but smoother release rates at low pH is especially beneficial for combination therapeutic drug delivery, where the acidic environment in the tumor tissues and late endosomes/lysosomes in cells facilities drug release from IO-MMMs, while the release in blood and other normal tissues are slow reducing unwanted side effects. Furthermore, the initial burst release of drugs is preferable to achieve a sufficient initial drug dosage in tumor treatment, with the sustained release necessary to prevent further cancer proliferation. Similar pH-dependent drug release profiles with various nanomedicines have demonstrated better therapeutic and antitumor effectiveness in delivering chemotherapeutic drugs when tested both *in vitro* and *in vivo*^19,76,78,80^. It is worth pinpointing that particles where drug molecules are covalently conjugated to their surfaces usually exhibit much lower drug loading efficiencies and more difficulty in drug release due to the covalent binding. Thus, the prepared drug@IO-MMMs with the high drug payload capacity and the excellent pH-dependent drug release properties, are both essential requirements to attain a notable level of therapeutic dose at the target cancerous cells and tumor tissues, resulting in effective cancer therapy.

### Biological and cytotoxicity assays

**Figure 6:**
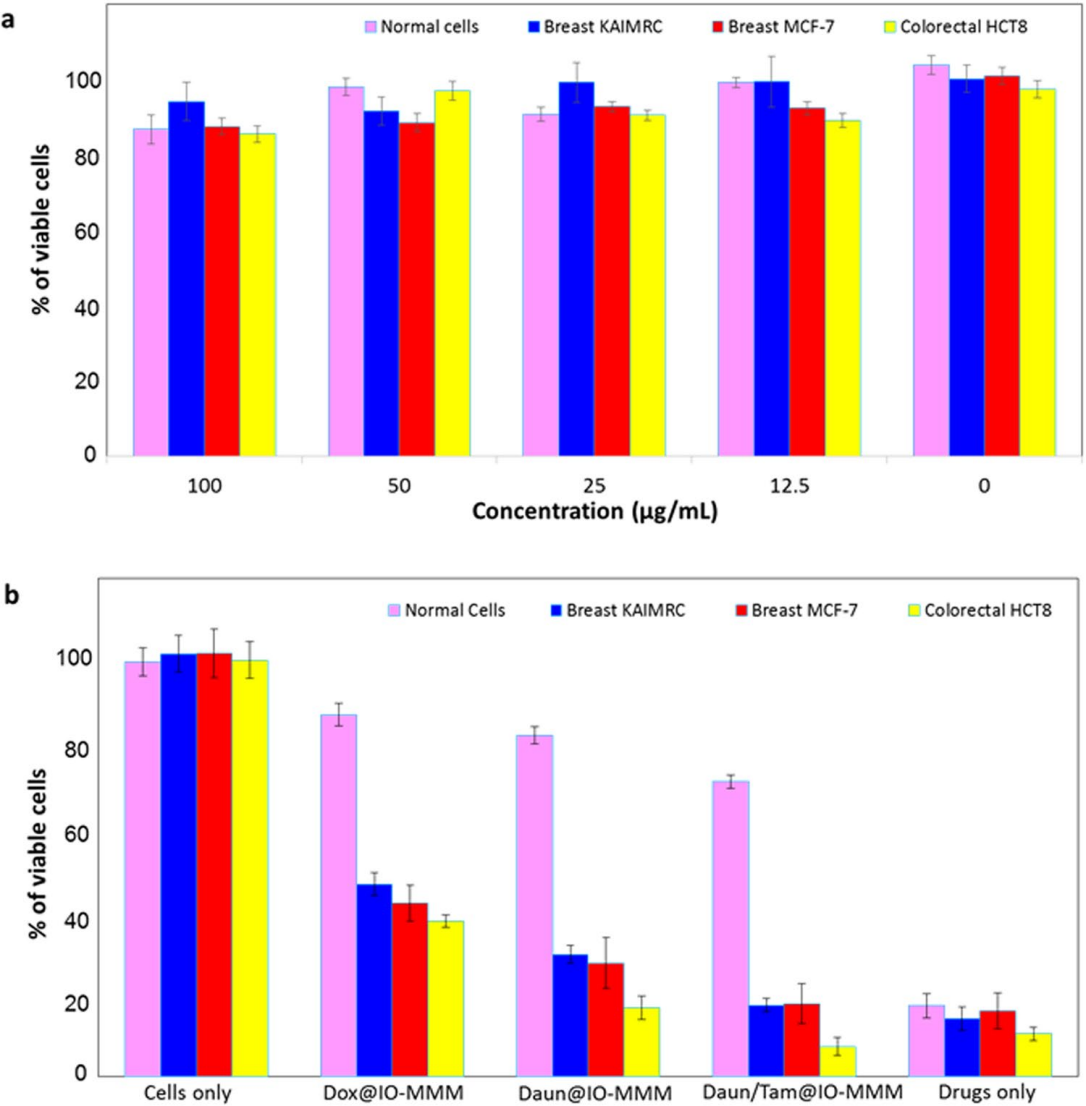
MTT cell viability assays for drug-free IO-MMMs and drug-loaded IO-MMMs with different types of cancerous and primary normal cells. (**a**) Percent of viable cells upon incubation with different concentrations of IO-MMMs up to 100 *μ*g/mL as determined by MTT cell viability assay. (**b**) MTT for the three different human cancerous cell lines along with normal primary cells treated with different types of drug@IO-MMMs. The results clearly show the selectivity of drug@IO-MMMs towards primary cells compared to cancerous cells, where the combinatory Daun/Tam@IO-MMMs showed up to 10 fold increase in potency for the colorectal HCT8 cell line compared to the primary normal cells. The experiments were carried out in triplicates and error bars denote standard deviations.

The cytotoxic effects of IO-MMM and drug@IO-MMM formulations towards different types of human breast (MCF7 and KAIMRC1) and colorectal (HCT8) cancerous cells, as well as normal primary cells were then examined using the thiazolyl blue tetrazolium bromide (MTT) cell viability assay. First to demonstrate the potential utility of IO-MMMs as biocompatible delivery vehicles, the as-prepared IO-MMMs were tested against the different cancerous cells. IO-MMMs elicited no cytotoxic effects to any of the tested cells, even at high concentrations up to 100 µg/mL particles (Fig. 6a). Importantly, the different drug-loaded IO-MMM formulations (30 µg/mL particles; 7.5 µg/mL for Dox or Daun and 5.25 µg/mL Tam) were found to be highly toxic to MCF7 and KAIMRC1 breast cancer cells as well as to HCT8 colorectal, with the least potency towards the normal primary cells (Fig. 6b). Interestingly, the combinatory Daun/Tam@IO-MMM formulation showed the most potent effects on the different cancerous cells tested with 10-fold and 5-fold enhanced cytotoxicities against the metastatic breast cancer and colorectal cancer cells in comparison to normal cells, respectively. It is anticipated that upon internalization, IO-MMMs release both Daun and Tam inside the cells, and, hence, enhance the cytotoxic effect through exhibiting synergistic anti-proliferative activities on the different cancerous cell lines. On the other hand, the free drugs, at equivalent concentrations, were found to be concurrently toxic to all the tested cells. This is in agreement with our previously published reports^72,81^. While the potency of drug@IO-MMMs towards the different cancerous cell lines was comparable to the potency of free drugs, it significantly decreased against the normal primary cells. This observed enhanced cytotoxicities of drug@IO-MMMs compared to free drugs towards cancerous cells, with the least sensitivity towards the normal cells suggest huge potentials for IO-MMMs as efficient and selective drug delivery vehicles.

**Figure 7:**
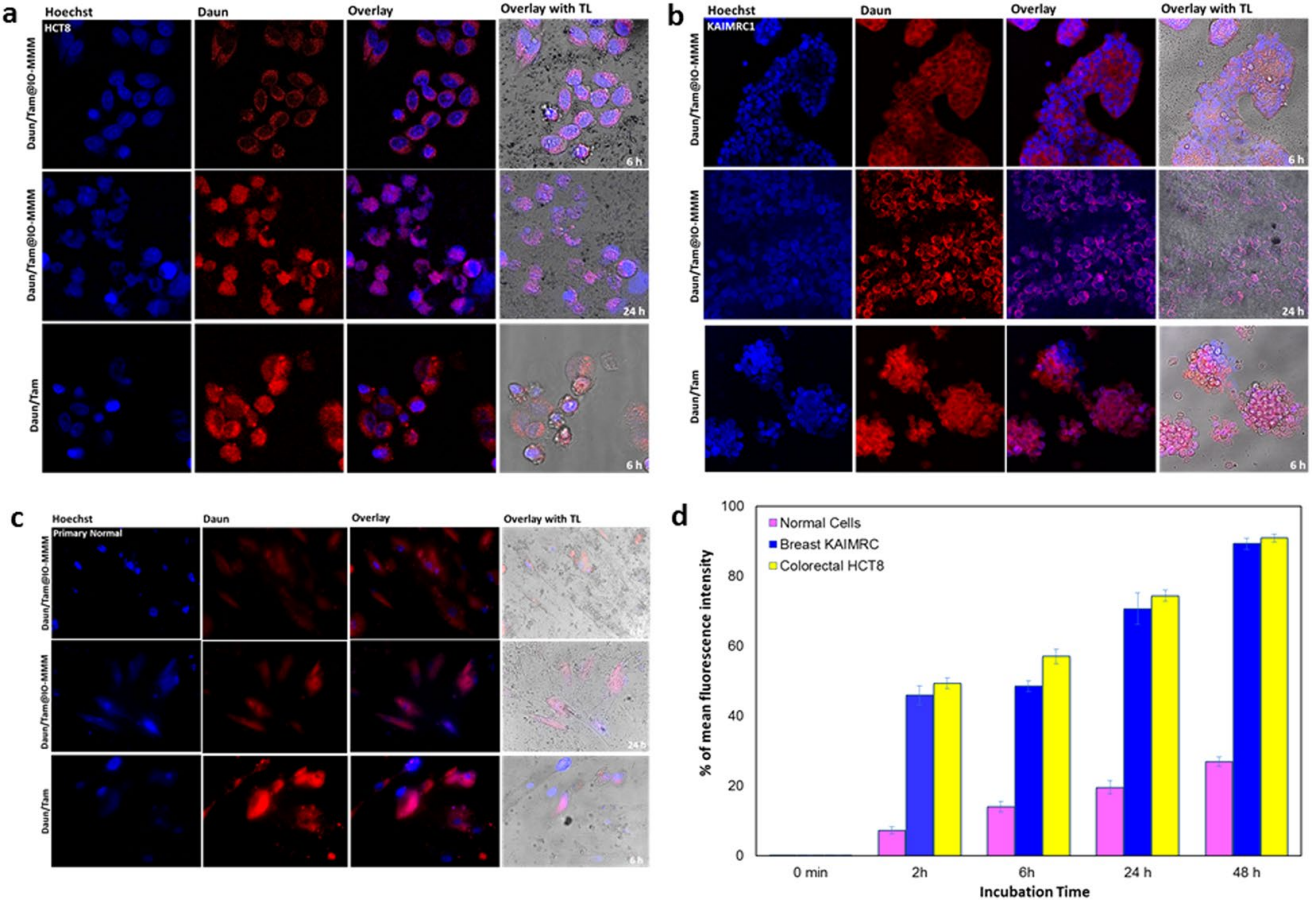
Live confocal microscopy images of (**a**) colorectal HCT8, (**b**) breast KAIMRC1, and (**c**) primary normal cells treated with Daun/Tam@IO-MMMs (10 μg/mL particles; 2.50 μg/mL Daun; 1.75 μg/mLTam) or equivalent concentrations of free Daun/Tam. Left to right: Hoechst channel (blue) showing nuclei; Daun channel (red); overlay of both channels; and overlay of Hoechst and Daun along with transmitted light (TL). The images of incubated cells clearly indicate that while the free drug diffused directly to the nuclei of all cells causing death even after 6 hrs, Daun/Tam@IO-MMMs-treated cancerous cells showed red signal mostly in the cytoplasm, increasing gradually in intensity and translocating to the nucleus with time causing apoptotic cell death (typical apoptotic features such as condensation and irregular membrane blebbing are clearly seen in the overlaid images). For primary normal cells, however, the red fluorescence was only apparent in the cytoplasm, with no significant apoptotic features observed. (**d**) Flow cytometry analysis of the cellular association of IO-MMM uptake by the different tested cells after incubations up to 48 hrs. The intracellular uptake was much higher for HCT8 and KAIMRC1, with the least uptake observed for the normal cells. Data are presented as means ± standard deviations.

To explore the route and intracellular drug delivery of the drug@IO-MMM constructs to the different cell lines, confocal laser scanning microscopy (CLSM) was performed. To mimic physiological conditions, live confocal imaging with no fixation of cells was conducted. We treated two different types of cancerous cells (i.e. colorectal HCT8 and breast KAIMRC1) along with primary normal cells with IO-MMMs, Daun/Tam@IO-MMMs or equivalent concentrations of the respective free drugs. All the cells treated with control IO-MMMs appear healthy with clear blue Hoechst nuclei stains and no apoptotic features or death observed (Supplemental Fig. S6). In the drug-treated cells, however,the distribution of the red Daun fluorescence showed a pattern that varied for the different cells exposed to free Daun/Tam *vs *Daun/Tam@IO-MMMs. In Daun/Tam@IO-MMMs-treated cancerous cells, confocal images confirmed that Daun delivered by Daun/Tam@IO-MMMs is entering the cells gradually as time elapses causing apoptotic cell death after 24 hrs (Fig. 7). The intensity of red fluorescence increased progressively in cells with incubation time, resulting from the sustained drug release property of drug@IO-MMMs. While typical apoptotic features were clearly evident for HCT8 and KAIMRC1 cells, the primary cells appeared to be healthy even after 24 hrs of treatment. Head-to-head comparison between the three cell lines treated with Daun/Tam@IO-MMMs after 24 hrs showed that Daun was translocated to the nucleus in cancerous cells, with more killing observed for HCT8 compared to KAIMRC1 cells, confirming the cell viability cytotoxicity results (Fig. 7a,b). On the other hand, normal primary cells showed the least florescence intensities, where Daun was found to be mainly in the cytoplasm, with no prominent red nuclear staining or apoptotic features detected (Fig. 7c). Importantly, when incubating the same cell lines with free Daun/Tam at equivalent concentrations, the red fluorescence was found to be directly localized in the nucleus of all cells concurrently with minimal detectable presence in the cytoplasm, even after only 6 hrs of incubation.

### Cellular internalization of IO-MMMs

Next, in order to gain insights into how the IO-MMMs interacted with the cells, their uptake with the different tested cell lines was examined by flow cytometry. Upon incubation of the cells with fluorescent-labeled IO-MMMs (10* μ*g/mL), all cell types showed significant cellular uptake gradually increasing with time, but slowly declining after 24 hrs (Fig. 7d). Interestingly, the fluorescence signals of cancerous cells demonstrated an uptake approximately 7-fold and 4-fold higher at 2 hrs and 24 hrs of incubation, respectively, compared to the primary normal cells with the least uptake. This confirmed and explained the confocal microscopy results obtained. Our findings are consistent with the results of previous studies by us^72,73^ and others^82,83^, reporting on the increased uptake of IONPs by human breast, leukemia and cervical cancer cells compared to normal cells. While this definitely requires further mechanistic studies, it is mostly attributed to the variations in cell-membrane compositions, characteristics, morphologies, immunoprofiles, and metabolic activities between the tested cancerous and normal cells^82–85^. The high metabolic activity of tumor cells along with the low metabolic activity of primary normal cells can particularly contributes to the observed increase in IO-MMMs’ uptake. Even when the normal primary cells grew in culture dish, they acquire much more time to confluent with double the amounts of growth factors added.

**Figure 8:**
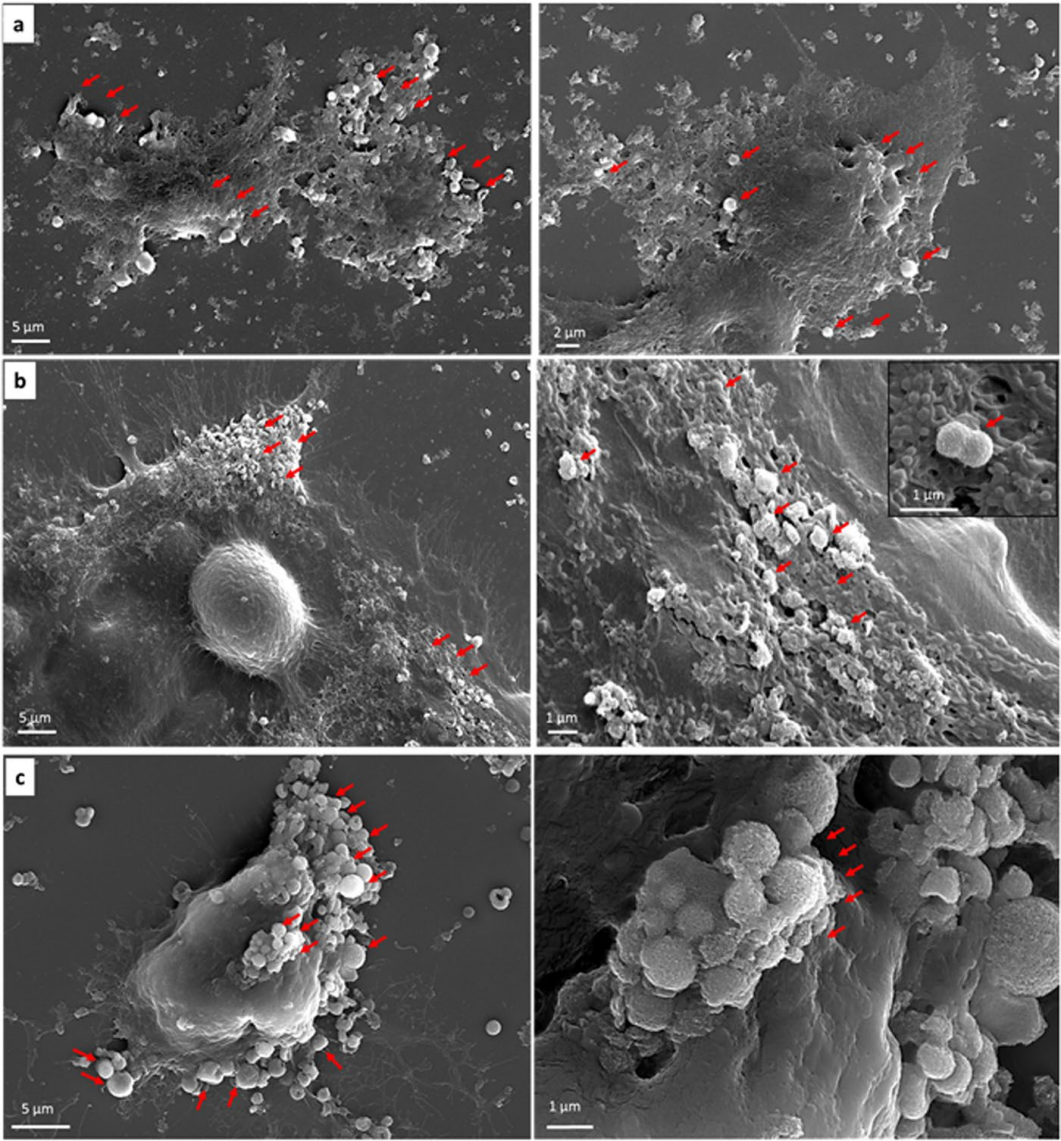
SEM micrographs of the different cancer cells after treatment with IO-MMMs at different magnifications. (**a**) Colorectal cancer HCT8 cells; (**b**) Breast cancer KAIMRC1 cells; (**c**) Breast cancer MCF7 cells. IO-MMMs (depicted in red arrows) were found to be cell-associated, attached to the periphery, and internalized inside. Image (**c**) clearly shows the attachment of IO-MMMS on the cell surface and membrane wrapping around IO-MMM for internalization.

**Figure 9:**
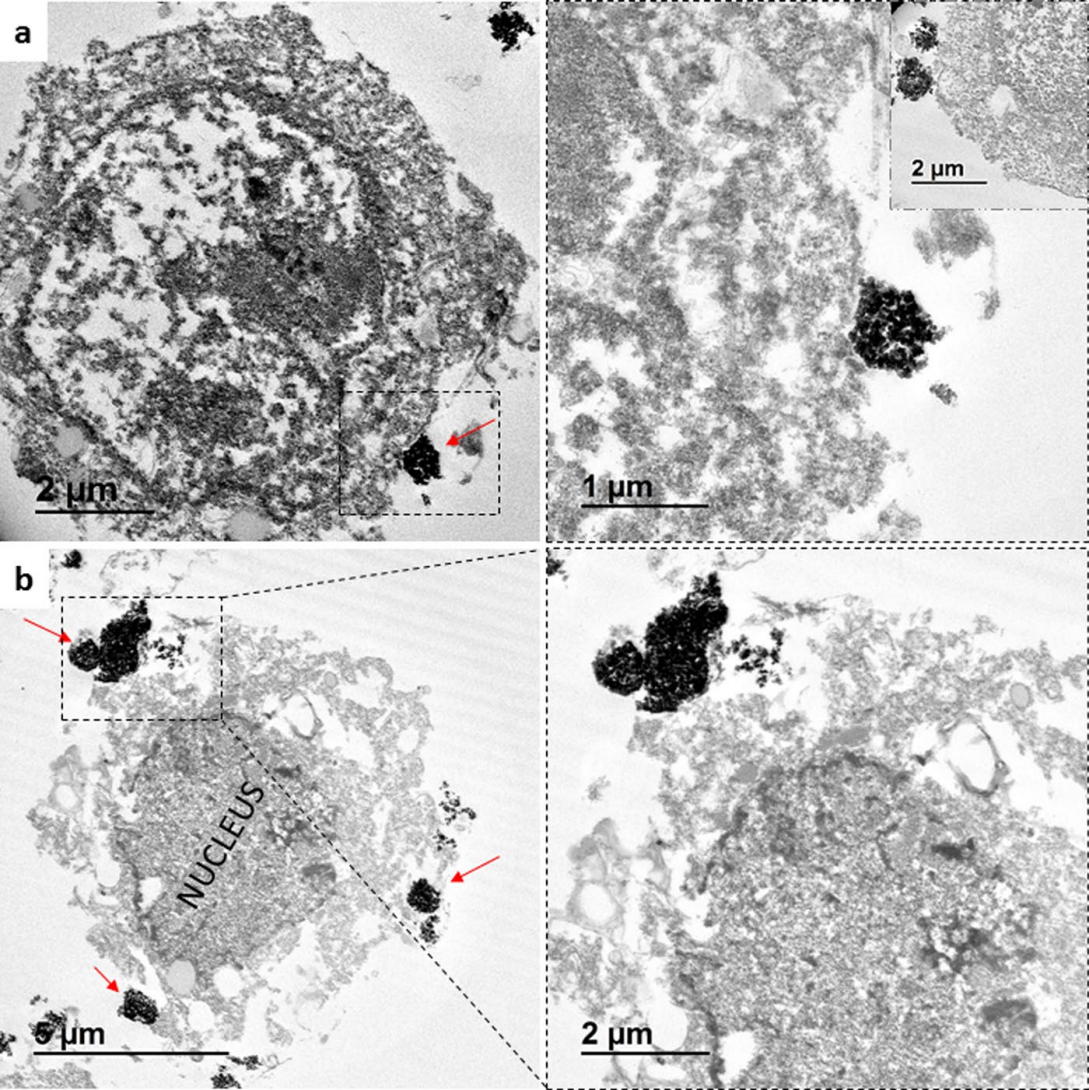
TEM images of representative cancer cell line treated with IO-MMMs at different magnifications. The micrographs clearly show (**a**) the attachment of the particles (shown in red arrows) to the plasma membrane, and (**b**) their subsequent internalization inside the cytoplasm. Note the cell membrane-particle engulfment involves microvilli where the cell protrudes its pseudopodia to catch the particles and move them inwards indicative of endocytic uptake. No particles were found to be located in the nucleus.

To further verify the uptake of the IO-MMM-treated cells at the sub-cellular level, the intracellular distribution was evaluated by electron microscopy imaging. SEM images clearly indicate that the tested cells uptake the particles mainly found to be attached to the cellular membranes and consequently internalized inside (Fig. 8a). Some IO-MMMs were found to be located on the periphery of the cells attached to their surfaces (Fig. 8b). However, wrapping of the cellular membrane around the particles is clearly evident (Fig. 8c) proving that the IO-MMMs are cell-associated, interact with the plasma membrane, and are subsequently internalized in clusters. To validate the intracellular location, TEM imaging was also employed. After only 2 hrs of IO-MMM cell incubation, particles were seen interacting with plasma membrane, microvilli, and within cells (Fig. 9a,b). Representative TEM micrographs clearly show that the IO-MMMs are attached and then internalized, individually or in clusters, by the cells *via* the plasma membrane. Despite their relatively larger sizes, the particles were majorly seen internalized in the cytosol, but not in the nucleus, in agreement with previous reports^67^. The particles’ engulfment took place without the particles being surface functionalized by polymeric or targeting groups, consistent with earlier reported works where particles up to 1000 nm were found to be abundantly present in different areas of the cytoplasm^86,87^. Commonly, cells uptake particles through one form of endocytic mechanisms, engulfed within the cell membrane, and contained intracellularly in membrane-bound vesicles which then traffic *via* endolysosomal pathways^88^. Studies have revealed that the particles’internalization, and their subsequent intracellular routing, is strongly dependent on their size, shape, composition, and surface properties, as well as on cell-specific parameters such as cell type, protein expression levels, or cell cycle phase^89,90^. The size-dependent interaction of different particles with the cell membrane is likely related to the membrane-wrapping process that initiates endocytosis, as has been observed here. Despite huge efforts in this area, it remains challenging and ambiguous to reliably correlate a particular cellular response with particle size. However, it is generally accepted that larger particles (>500 nm) are predominantly taken up *via *nonspecific energy-dependent membrane-tangled internalization and macropinocytosis^91^. Moreover, the observed uptake may be possibly due to the high adsorption of phospholipid head groups on the plasma membranes to surface hydroxyl groups (Fe-OH) *via* a proven Fe–O–H–O–P mechanism^92^. We, thus, anticipate the internalization to occur by a combination of mechanisms, most notably non-selective macropinocytosis^93^, where internalization of upper-size-limit particles up to 3-μm was elegantly demonstrated^90^. After being uptaken, the particles subsequently traffic to lysosomes to be hydrolyzed and broken down. Interestingly, in some micrographs it was possible to observe agglomerates of iron oxide clusters closer to the nucleus, suggesting their traffic to acidic lysosomal compartments (Supplemental Fig. S7). It is worth noting that no major alternations in cellular structures or micro-organelles was apparent, confirming the biocompatibility of the utilized IO-MMM carriers. As compared to particles with smaller sizes (<200 nm), although the net internalization might be less, the actual drug release into the cytosol may well be higher, due to the prolonged residence time of the microparticles inside, thus avoiding rapid lysosomal degradation^91^. Another important benefit, is that microparticles cannot passively diffuse into cells, enter organelles (i.e. mitochondria), and disrupt normal cell functions, which thus minimizes potential toxic and systemic effects of smaller nanoparticles after administration^94^. According to our knowledge, this is the first report systematically studying the intracellular uptake of iron oxide mesostructures of sizes (>500 nm).

Collectively, all the results imply that the major route of drug delivery is through differential uptake of drug@IO-MMMs into the cells, followed by sustained release of drugs intracellularly in a pH-dependent manner, causing apoptotic cell death. It is, thus, repeatedly evident that while free drugsare internalized by passive diffusion, the uptake of iron oxide nano/microparticles are rather directed by endocytic trafficking mechanisms. Regardless of the exact and complex intracellular routing, our results strongly suggest that using the IO-MMM carriers described here is superiorly advantageous, as it reduces the unwanted diffusive side effects of the free drugs allowing selective synergistic anticancer drug delivery.

### Penetration of drug@IO-MMMs into tumor tissue

In order to investigate the penetration of Daun/Tam@IO-MMM formulation into tumor tissues, we treated primary breast tumor surgery sections derived directly from primary lesions with either Daun/Tam@IO-MMMs or free Daun/Tam at equivalent drug concentrations. Due to inadequate drug accumulation, tumor drug resistance, and limited drug penetration of chemotherapeutic drugs to tumor tissues^95^, it is imperative to design advanced delivery systems to enhance tumor accumulation, improve drug delivery, and, hence, enhance overall efficacies^96^. While free drugs diffuse nonspecifically with typically limited penetration to interior of tumors, drug delivery carriers have premises to better accumulate in the tumor tissues *via* the acknowledged enhanced permeability and retention (EPR) effect and kill the cancer cells residing in the center, resulting in enhanced therapies^4,11,97^. If anticancer drugs are unable to access and penetrate the cells within a tumor, then their therapeutic effectiveness will be hampered, no matter what is their actual potency^98^. Despite the existence of different strategies for stimuli-responsive triggered drug release systems (i.e. pH, redox, enzymes, heat, light etc) intended to better penetrate tumors, many barriers still exist^16,99^. In fact, the tumor penetration is a passive process that requires a bio-stable particles of appropriate sizes (not too small nor too big) loaded with high capacities of drugs to allow extravasation of the particles across the hyper-permeable tumor vessels and effective diffusion through the tumor interstitial space^100^. Primary tumor vessels have a defective cellular lining composed of disorganized, loosely connected, overlapping or sprouting endothelial cells where the gaps have been reported to range between 300 nm and 4700 nm^101,102^. This particularly contributes to tumor vessel leakiness and permits access of therapeutic vehicles. The challenge remains to deliver the particles throughout the tumor and its metastases given the heterogeneity of tumor microenvironment along with the limitations of spatial and temporal changes in the expression of the target. One of the most promising directions is, thus, to employ an internal pH-dependent sustainable delivery system that accumulates in the tumor and delivers drug-loaded particles into the target cells, releasing their loads intratumorally. Although nano-sized materials have been advocated for drug delivery to tumors, micron-sized particles such as Dox-loaded APMS have also shown promise in enhancing intracellular uptake of Dox and inhibiting tumor growth without any systemic toxicity both *in vitro* and *in vivo*^103^. Therefore and based on the promising *in vitro* cellular studies obtained, we anticipate that the pH-triggered IO-MMM formulation designed here, will attain the above-mentioned criteria.

**Figure 10:**
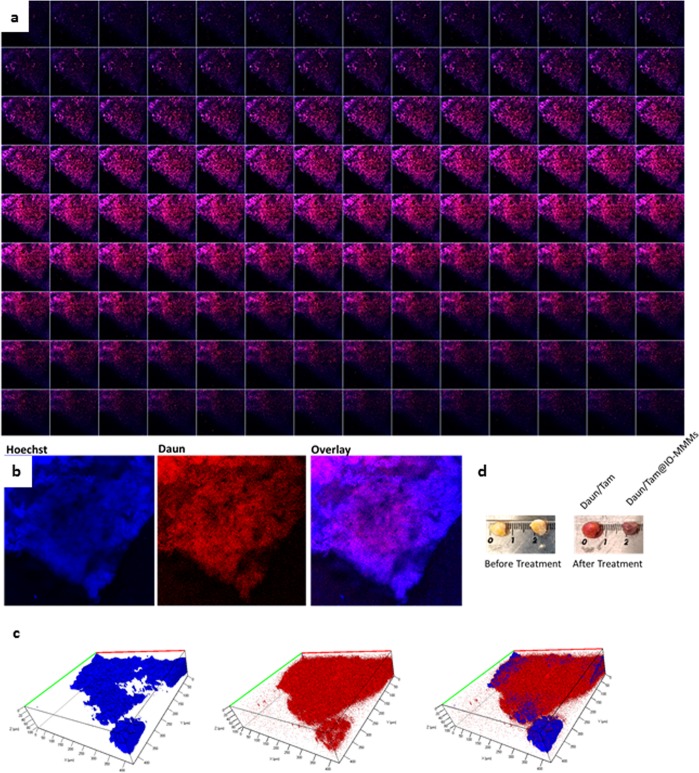
(**a**) Gallery view of z-stack images of a patient breast cancer tumor tissuetreated with Daun/Tam@IO-MMMs for 2 weeks at 37 °C and 5% CO_2_. EC Plan-Neofluar 10×/0.30 objective was used to image this stack with a resolution of 512 × 512. Pixel dwell time was 1.58µs and z-dimension was divided into 50 optical slices from top to bottom. (**b**) Representative middle slice image of the z-stack images: Hoechst channel (blue); Daun channel (red); and merged overlay of both channels. (**c**) 3D reconstruction of the confocal z-stack images. This is the projection reconstructed from the 50 optical sections using averaging projection method. Images clearly show the penetration of Daun delivered by IO-MMM vehicles to ~105 μm depth inside the tumor tissue. (**d**) Optical camera photographs of the tumor tissue before and after treatment. Even with the naked eye, the shrinkage of Daun/Tam@IO-MMMs-treated tumor tissue compared to free drug-treated tissue is obvious, confirming the potential of IO-MMMs as effective drug delivery vehicle penetrating deep inside the tumors improving the recognized limited penetration of free drugs to solid tumors.

Dissected breast cancer tumor tissues were cut to ~1 mm^3^ of equivalent sizes and were cultured to mimic oxygen, nutrient, and energy gradients similar to those found *in vivo*. The treatment was performed for 2 weeks at 37 °C and 5% CO_2_. Z-stack confocal microscopy images starting from top to bottom of patient biopsy tissue sample exposed to Daun/Tam@IO-MMMs were acquired (Fig. 10a). As shown, extensive distribution of red Daun fluorescence through the tumor from top to bottom was observed (a middle selected slice of the z-stack images is displayed in Fig. 10b). 3D reconstructed images excitingly showed areas of extensive red fluoresence deep inside the tissue(~105 μm depth), signaling significant intracellular Daun release inside the tumor (Fig. 10c). It is anticipated that the particles penetrated inside the leaky vasculature of tumor tissue to certain level, releasing the drugs internally. In comparison, the amount of red fluorescence penetration was noticeably much lower for the free drug (Supplemental Fig. S8). Thus, form our results, it is clear that the Daun/Tam@IO-MMM formulation penetrated deeper and delivered enhanced anticancer drugs into the tumor compared to the free diffusive drugs. Furthermore, optical photographs of tissue sections before and after treatment were also captured to register the shrinkage of tissue mass due to the different treatments (Fig. 10d). Even with the naked eye, the shrinkage of Daun/Tam@IO-MMMs-treated tumor tissue compared to free drug-treated tissue was obvious, confirming the potential of IO-MMMs as effective drug delivery vehicle penetrating inside the tumor *via* the EPR effect, and significantly improving the recognized limited tumor penetration of free drugs. It is to be noted that similar tumor tissues were also treated with either Daun@IO-MMMs or Dox@IO-MMMs alone, however, lower therapeutic effects were observed, signifying that the co-delivery of Daun and Tam together synergistically exert their anticancer functions to achieve a better outcome.This designed multi-drug IO-MMM formulation is, thus, promising to enhance the effectiveness of the therapeutic drugswith optimal doses, opening new opportunities for *in vivo* combined therapeutics. This combinatory drug therapy depicts the synergistic enhanced antitumor effectswhere lower doses ofeach therapeutic drugs can be used, which in turn, will have a dramaticeffect in cancer treatments, minimizing the typical toxicity towards the healthy cells. Furthermore, magnetic guidance on this system is potentially feasible by focusing an external magnetic field on the target after injection of IO-MMMs.

## Conclusion

In summary, unique bio-stable iron oxide mesoconstructs with remarkably high surface areas and pore volumes were prepared by hard-templated methodology using APMS as the silica template. The as-prepared IO-MMM mesostructures can be loaded with hydrophilic and hydrophobic therapeutic anticancer drugs, or a combination of both drugs, on demand. The combinatory drug@IO-MMM formulation demonstrated cellular uptake and pH-dependent drug release with enhanced cytotoxic effects towards different types of cancerous cells. Particularly, Daun/Tam@IO-MMMs were found to be highly potent to the different metastatic breast and colorectal cancer cells, with considerably less toxicity towards the normal primary cells, suggesting huge potentials as selective anti-cancer agents for cancer therapeutics. Importantly, this drug-loaded IO-MMM formulation was also able to effectively accumulate in patient primary tumor tissues, delivering the cytotoxic drugs and killing the tumor cells inside. The Daun/Tam@IO-MMM vehicle described here facilitates packing of chemo/hormonal therapeutic anticancer drugs without premature release at physiological pH, recognizes the target cells, and finally releases the drugs at the intracellular site of tumor cells to induce apoptosis and shrinkage. Notably, this combinatory mesoporous drug@IO-MMM platform display not only a pH-dependent but potentially a magneto-responsive drug release behavior, opening new premises for *in vivo* imaging, cancer monitoring, and theranostics.

## Materials and Methods

Unless otherwise indicated, all chemicals and solvents were obtained from commercial suppliers and used as received without further purification. Iron (III) chloride hexahydrate (FeCl_3_·6H_2_O), zinc metal powder, magnesium powder, cetyltrimethylammonium bromide (CTAB), tetraethyl orthosilicate (TEOS),3-aminopropyltriethoxysilane (APTES), sodium fluoride (NaF), concentrated HCl, ammonium hydroxide (NH_4_OH), iron (II) chloride tetrahydrate (FeCl_2_.4H_2_O), polyacrylic acid (PAA), fluorescein isothiocyanate(FITC) as well as the drugs Doxorubicin (Dox), Daunorubicin (Daun), and Tamoxifen (Tam) were all purchased from UFC Biotechnology. Dulbecco’s Phosphate Buffered Saline (DPBS), Phosphate Buffered Saline (PBS), Advanced Dulbecco’s Modified Eagle Medium (DMEM), Phenol-red free DMEM, Fetal Bovine Serum (FBS), Hoechst 33342 stain, L-Glutamine, and Penicillin-Streptomycin (Pen-Strep) were all purchased from Invitrogen. MTT (Thiazolyl Blue Tetrazolium Bromide) powder was purchased from Bioworld, USA. All cell lines were purchased from the American Type Culture Collection (ATCC) and grown in Advanced DMEM supplemented with 10% FBS and 1% Penicillin/Streptomycin. Human cancerous cells used in this study are: MCF7 (Michigan Cancer Foundation-**7** metastatic breast cancer cell line isolated from a 69-year-old Caucasian woman); HCT-8 (Human ileocecal colorectal adenocarcinoma cell line isolated from a 67-year-old male); KAIMRC1 (naturally immortalized KAIMRC1 breast cancer cells isolated from a 62-year-old Arab female suffering from stage IIB breast cancer); along with primary normal fibroblast-like cells (isolated from a breast cancer patient after surgical procedure) used as a control. Cells were cultured for at least 24 hrs before conducting the experiments. All experiments were conducted in triplicates and mean averages were plotted. Tumor surgical sections were collected after examination by a certified pathologist, prepared for analysis on the same day, and cultured in advanced DMEM. Tissues were collected at the time of surgery after written informed consent was obtained from patients at King Abdulaziz Medical City, National Guard Hospital, under approval from KAIMRC Institutional Review Board (IRB). The tissue biopsies were surgical remnants which were consequently used in this study. All methods were performed in accordance with standard guidelines and regulations, and the procedures of tissue experiments were approved by Bioethics Department, IRB, KAIMRC (RC16/096).

### Characterization

SEM images were processed usinga FEI NanoSEM 450 scanning electron microscope at 15 kV. EDX were acquired using same system which is supplied with EDAX® AMETEK® (material analysis division). TEM images were collected on a JEOL-JEM 1400 operating at 120 kV using Gatan camera with Digital Micrograph Imaging software. Samples were prepared by depositing 1 μl of the nanoparticles dispersed onto 400 mesh Formvar/Carbon-supported copper grid. X-ray diffraction (XRD) patterns were recorded using Bruker D8 Diffractometer using Cu Kα radiation operated at 40 kV and 2 Ɵ scan range from 20° to 70°. FTIR spectra (400–4000 cm^−1^) were recorded as KBr pellets using Shimadzu IRAffinity-1. DLS and zeta potential measurements were assessed on Malvern Zetasizer Nano ZS instrument. Calcinations were carried out either under flowing air or under argon. The following heating profile was used for APMS calcination: 2 °C/min ramp to 450 °C, 240 min hold at 450 °C, 10 °C/min ramp to 550 °C, 480 min hold at 550 °C. Confocal images were visualized using inverted Zeiss LSM 780 multiphoton laser scanning confocal microscope equipped with 20× and 40× (oil immersion) objectives and Axiocam cameras. Z-stack images of tissue sections were acquired using above mentioned Zeiss LSM780 microscope.

#### Preparation of APMS

APMS was synthesized following our previous work where TEOS was used as the silica source and CTAB as the structure directing agent. Briefly, CTAB (0.7 g), H_2_O (15 mL), ethanol (5 mL), and concentrated HCl (1.7 mL) were vigorously stirred until the surfactant was dissolved. TEOS (1.4 mL) was then slowly added while stirring. After 5 min, NaF (0.5 M in water, 1.85 mL) was added and stirring was continued until the mixture turned turbid. The mixture was then quickly transferred to a Teflon bottle and heated at 100 °C for 60 mins. The resulting APMS precipitate was cooled to room temperature, collected by vacuum filtration, repeatedly washed with water and ethanol, and dried under vacuum. Calcination at 550 °C under air to remove the CTAB surfactant afforded calcined APMS ready for iron impregnation.

#### Preparation of IO-MMMs

IO-MMMs were prepared following modified literature procedures^39,62^ using calcined APMS as the hard template. 0.175 g of APMS was dispersed in 10 mL of 0.5 M aqueous solution of FeCl_3_.6H_2_O. Trace amounts of zinc (or magnesium)was then added and the reaction mixture was aged for 24 hrs under constant stirring at room temperature. The resulting material was carefully decanted to separate the zinc, purified by successive centrifugation and repeated washing with water and ethanol, and finally dried under vacuum. This impregnation process was then repeated with 0.5 M of FeCl_3_ aqueous solution to ensure complete iron impregnation. The obtained product was then heated to 350 °C at 2 °C/min for 3 h under argon atmosphere and left at this temperature for 4 h. Thermal treatment at 350 °C under inert atmosphere is adequate to completely decompose and convert iron species to *γ*-Fe_2_O_3_ nanocrystals, without formation of other phases. Finally, the sample was treated several times with 5 M hot NaOH solution to completely remove the APMS silica template, purified by centrifugation, washed with water and ethanol, and then dried in an oven at 100 °C. IO-MMMs were dispersed in water for cellular studies. It is worth pinpointing that different FeCl_3_ aqueous solutions (0.25, 0.5, 0.75, and 1 M) for impregnation were tested. No much difference was observed when greater than 0.5 M solutions were used. This experimental procedure can be extended to a wider range of binary transition metal oxides by simply using a wide range of the commercially available cheap metal salt precursors.

#### Preparation of IO-MMNs

First, PAA-MNPs were prepared according to our previous report^72^. In a generalized procedure, 5 mL aqueous solution of FeCl_3_.6H_2_O (0.10 g) and PAA (0.10 g) were mixed and magnetically stirred under argon in a degassed vial for 15 min. Aqueous solution of FeCl_2_.4H_2_O (0.05 g) was then injected. After stirring for a few minutes, 1 mL of ammonium hydroxide (30%) was slowly added and the mixture was heated to 80 °C and vigorously stirred for 90 min. The black precipitate of PAA-MNPs formed was cooled to room temperature by removing the heat source, isolated *via* centrifugation (4500 rpm, 10 min), washed repeatedly with water, isopropanol, and ethanol, and finally redispersed in water. Centrifugation (4500 rpm, 20 min) was then applied to remove any undispersed residue. The stable colloidal PAA-MNPs solution was stored at ambient conditions for several months. No precipitation over the course of months was observed. It is to be noted that when hexylamine (0.5 mL) was added in the synthesis, PAA-MNPs clumpand precipitate, and the particles were found to be dispersible in ethanol. 0.10 g of as-prepared calcined APMS was then impregnated/wetted with aqueous or ethanolic solution of PAA-MNPs (0.1 to 2 mg/mL) and the reaction mixture was stirred for 24 hrs at room temperature. The resulting material was then centrifuged, washed repeatedly with water and ethanol, dried under vacuum, and thermally treated as before. Etching using5 M NaOH solution afforded very low yields of IO-MMNs instead, with low BET surface areas and pore volumes (Supplemental Fig. S2). Even when repeated impregnation was performed, no much of a change in the final product was observed.

#### Magnetic Manipulation of IO-MMM Magnetized Cells

Prior to the experiment, cells were seeded on 35 mm Ibidi dishes for 24 h at 37 C and 5% CO_2_. Cells were then treated with IO-MMMs for 1 hr, washed with PBS, stained with HOECHST 33342 nuclear stain, and imaged using EVOS FL Auto Imaging System in the presence or absence of a permanent magnet. Both transmitted light and blue fluorescence images were acquired. Optical camera photos were also shot recording the aggregation of cells in the presence of the magnet.

#### Drug loading

1 mL aqueous dispersion of IO-MMMs(1.0 mg/mL) and aqueous drug solutions (250 μg/mL) were gently shaken on a rotary shaker for 36 hrs to enable maximal drug loading. The particle dispersions was then isolated *via* centrifugation (4500 rpm, 10 min), washed repeatedly with water until no drug was detected in the supernatant by UV-vis, and finally redispersed in water to form stable aqueous dispersions of drug@IO-MMMs. In case of Tam and Daun loading, Tam was first loaded followed by Daun to afford Daun/Tam@IO-MMMs (165 μg/mL Tam; 235 μg/mL Daun). Loading efficiencies were determined by UV-vis spectroscopy as depicted below. The absorbance of the residual drug in the supernatant was measured (λ_max_ = 490 nm for Dox and Daun; λ_max_ = 290 nm for Tam) and the percentage of drug loading (w/w%) was then quantified. The loading efficiency was calculated as:

$${\rm{Loading}}\,{\rm{Efficiency}}=({{\rm{W}}}_{{\rm{l}}}/{{\rm{W}}}_{0})\times 100$$

where W_l_ is the amount of drug loaded onto the particles and W_0_ is the amount of drug in the initial solution. The amount of drug loaded onto IO-MMMs was calculated from the difference between the initial drug concentration and the drug concentration left in the supernatant.

#### Drug Release

In a typical study, 5 mg of drug@IO-MMMs were suspended in 1 mL PBS buffer at two different pH values (pH = 7.4 and pH = 4.5) and gently rotated at 37 °C in an oven provided with a precise control in the temperature within 0.01 °C. The concentration of drugs in the supernatant was determined at fixed time intervals by UV-vis spectroscopy. At specific time points, supernatant aliquots obtained by centrifugation were taken from each tube, measured and returned to their respective tubes after UV-vis measurements. The drug concentration could be directly calculated from the measured absorbance. Triplicate aliquots were run for each time interval, and average values plotted. The percent drug release was calculated using the equation below:

$$ \% \,{\rm{Drug}}\,{\rm{Release}}=({{\rm{Abs}}}_{({\rm{t}})}\,{\rm{of}}\,{\rm{released}}\,{\rm{drug}}/{{\rm{Abs}}}_{({\rm{t}}0)})\times 100$$

#### Cell Viability Assays

All cell lines were seeded in a 96-well plate at a density of 5 × 10^4^ cells/well and incubated at 95%/5% humidified air/CO_2_ and 37 °C. Post 24 hrs, the media was removed and fresh phenol-red free DMEM containing 0.5% FBS was added to the cells. Cells were then treated with various concentrations of IO-MMMs, drugs@IO-MMMs, and respective free drugs in 200 μL supplemented DMEM. The wells at all edges were left free of cells in order to prevent edge effect. An additional row with only the particles was added in order to account for the particle effect. After 48 hrs of incubation, the media was removed and the cells were washed with PBS. Cell viability was then determined using the MTT viability assay following the manufacturer’s protocol. Briefly, 20 µl of MTT reagent (5 mg/mL) was added to each well and kept for 4 hrs at 37 °C in the incubator. The supernatant was then removed, and the MTT formazan were dissolved in 100 µl dimethyl sulfoxide (DMSO). The absorbance was measured on Molecular Devices SpectraMax microplate absorbance reader at 570 nm. The percentage of viable cells was calculated as the ratio of the absorbance of the treated group, divided by the absorbance of the control group, multiplied by 100. The absorbance from the untreated control cells was set as 100% viable.

#### Live Confocal Microscopy Imaging

Cells were incubated in 8-well dish ((iBidi, Germany) for 24 hrs prior to particle exposure. After removing the supernatant, cells were then exposed to IO-MMMs (10 μg/mL), Daun/Tam@IO-MMMs (10 μg/mL NPs; 2.50 μg/mL Daun; 1.75 μg/mL Tam), or equivalent concentration of free Daun/Tam. The cells were incubated for different periods of time (i.e. 6 and 24 hrs). Hoechst 33342 stain was then added. The cells were allowed to settle down for 20 min before microscopic visualization. To mimic physiological conditions, no fixation of cells was conducted.

#### Fluorescence Activated Cell Sorting (FACS)

Cancerous and normal cells (1 × 10^5^ cells per well) were seeded in 12 well culture dishes, incubated for 24 h, and then treated for different periods of time with FITC-labeled IO-MMMs (10 µg/mL) prepared following literature procedure^104^. Both live and dead cells were collected, pelleted, washed with PBS, and then transferred to FACS tubes for analysis. Flow cytometry measurements were then assessed. Triplicate experiments were conducted. Mean values were plotted and error bars represents standard deviations.

#### Scanning Electron Microscopy of Cell Specimens

Cells were treated with IO-MMMs (10 µg/mL) for 6 hrs prior to fixation. The specimens were then processed for SEM by the following method: Cells were first fixed with 4% paraformaldehyde at 4 °C for 30 min and then dehydrated with graded concentrations of ethanol. The cells were then transferred to appropriate carbon taped stubs (Ted Pella, USA) for imaging. To enhance the electron conductivity, samples were coated with gold/palladium (Au/Pd) by sputter coating and examined on a FEI NanoSEM 450 scanning electron microscope at 15 kV.

#### Transmission Electron Microscopy of Cell Specimens

Cells were first treated with the IO-MMMs for 2 hrs as described above. The specimens were then processed for TEM by the following method: the specimen was fixed in 4% glutaraldehyde in 0.1 M PBS (pH 7.4) for 1 hr. After washing in the same buffer, the sample was post-fixed in 1% OsO_4_ in 0.1 M PBS (pH 7.4) for 1 hr, followed by PBS washing. The specimen was then dehydrated in a series of acetone solutions and infiltrated in acetone: resin (1:1) for 1 hr, and then with acetone: resin (1:2) for more than 3 hrs. The specimen was then embedded in epoxy resin (Araldite) and placed in an oven at 80 °C overnight to polymerize. Ultrathin sections were obtained with Ultramicrotome (RMS), mounted on copper grids, and stained for contrast with heavy metal stains (uranyl acetate and lead citrate). TEM images were then collected on a JEOL-JEM 1200 operating at 120 kV using Gatan camera with Digital Micrograph Imaging software.

#### Surgical tissue processing

Human breastcancer patient tissue resections were placed in a sterile conical tube containing PBS on wet ice during transportation from the operating room to the research laboratory. Resections were transported to the laboratory within 30 min of clinical sample collection. Upon arrival, the tissue resections were examined by a certified pathologist. Resections were then manually minced using a sterile scalpel and washed thoroughly with PBS. Then small tumor pieces were transferred into 8-well µ-dishes (Ibidi, Germany) containing advanced DMEM supplemented with 10% FBS, 1% L-glutamine, and 1% antibiotics (Pen-Strep). Tumor tissue sections were treated with the different drug@IO-MMMs or equivalent concentration of free drugs for 7 days prior to imaging with an inverted Zeiss LSM 780 multiphoton laser scanning confocal microscope equipped with 20× and 40× (oil immersion) objectives and Axiocam cameras.

## Supplementary information

Preparation of iron oxide mesoporous magnetic microparticles as novel multidrug carriers for synergistic anticancer therapy and deep tumor penetration

## Data Availability

The data that supports the findings reported herein are available upon request from the corresponding author.
