# Supplementary material for: Preparation of iron oxide mesoporous magnetic microparticles as novel multidrug carriers for synergistic anticancer therapy and deep tumor penetration
Source: Sci Rep. 2019 Jul 1;9:9481. doi: 10.1038/s41598-019-46007-z (PMC6603044; doi:10.1038/s41598-019-46007-z)
Supplement: Supplementary file 1 — Preparation of iron oxide mesoporous magnetic microparticles as novel multidrug carriers for synergistic anticancer therapy and deep tumor penetration [file 41598_2019_46007_MOESM1_ESM.docx]

**Supplementary Information**

**Preparation of iron oxide mesoporous magnetic microparticles as novel multidrug carriers for synergistic anticancer therapy and deep tumor penetration**

Kheireddine El-Boubbou,^ab^* RizwanAli,^b^Hajar Al-Zahrani,^b^ThadeoTrivilegio,^b^Abdullah H. Alanazi,^c^Abdul Latif Khan,^c^Mohamed Boudjelal,^b^and AbdulmohsenAlKushi^a^

^a^Department of Basic Sciences, College of Science & Health Professions, King Saud bin Abdulaziz University for Health Sciences (KSAU-HS), King Abdulaziz Medical City, National Guard Health Affairs, Riyadh 11481, Saudi Arabia

^b^King Abdullah International Medical Research Center (KAIMRC), King Abdulaziz Medical City, National Guard Health Affairs, Riyadh 11426, Saudi Arabia

^c^Department of Pathology and Laboratory Medicine, King Abdulaziz Medical City, National Guard Health Affairs, Riyadh, 11426, Saudi Arabia

To whom correspondence should be addressed: E-mail: [elboubboukh@ngha.med.sa](mailto:elboubboukh@ngha.med.sa); [boubbouk@ksau-hs.edu.sa](mailto:boubbouk@ksau-hs.edu.sa)

**Contents:**

1. TEM images of IO-MMMs S2
2. Physiochemical properties of IO-MMNs S3
3. UV-vis and FTIR spectra of drug@IO-MMMs S4
4. Hydrodynamic size and zeta potentialsof drug@IO-MMMs S5
5. Schematic for drug release mechanism S6
6. Confocal images of the different cells incubated with IO-MMMs S7
7. TEM images of IO-MMM-treated cells S8
8. CLSM images of patient breast tumor tissue treated with free drugs S9

**
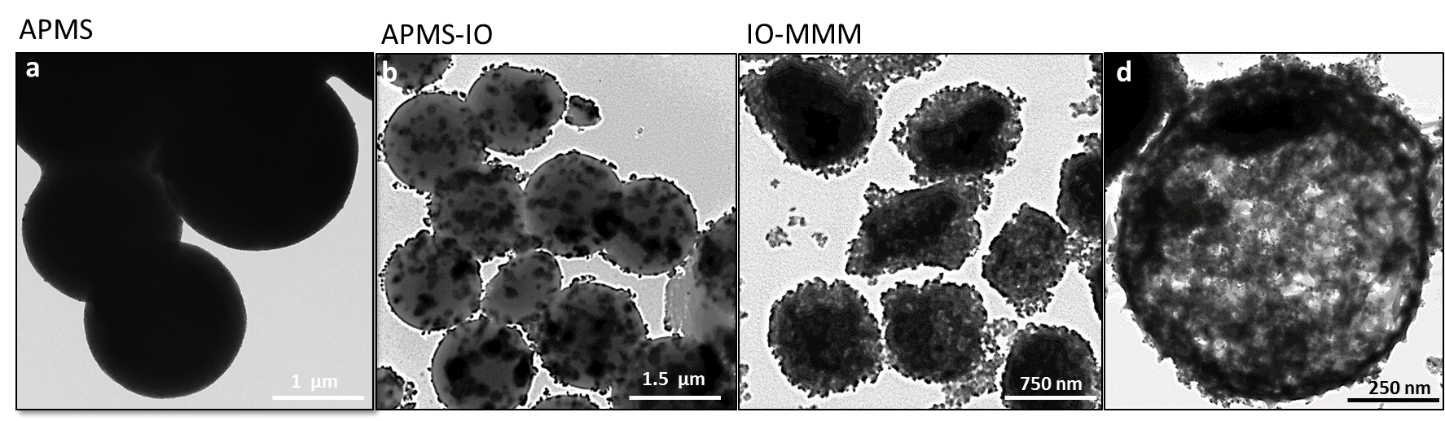
Figure S1.** TEM images of (a) APMS, (b) APMS-IO, and (c) IO-MMM showing the successful impregnation of iron oxide within the mesopores of APMS and subsequent formation of magnetic iron oxide mesostructures.

**
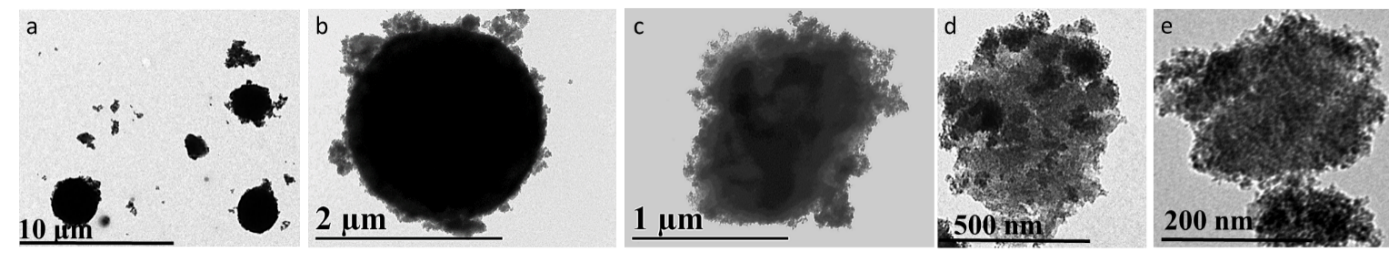

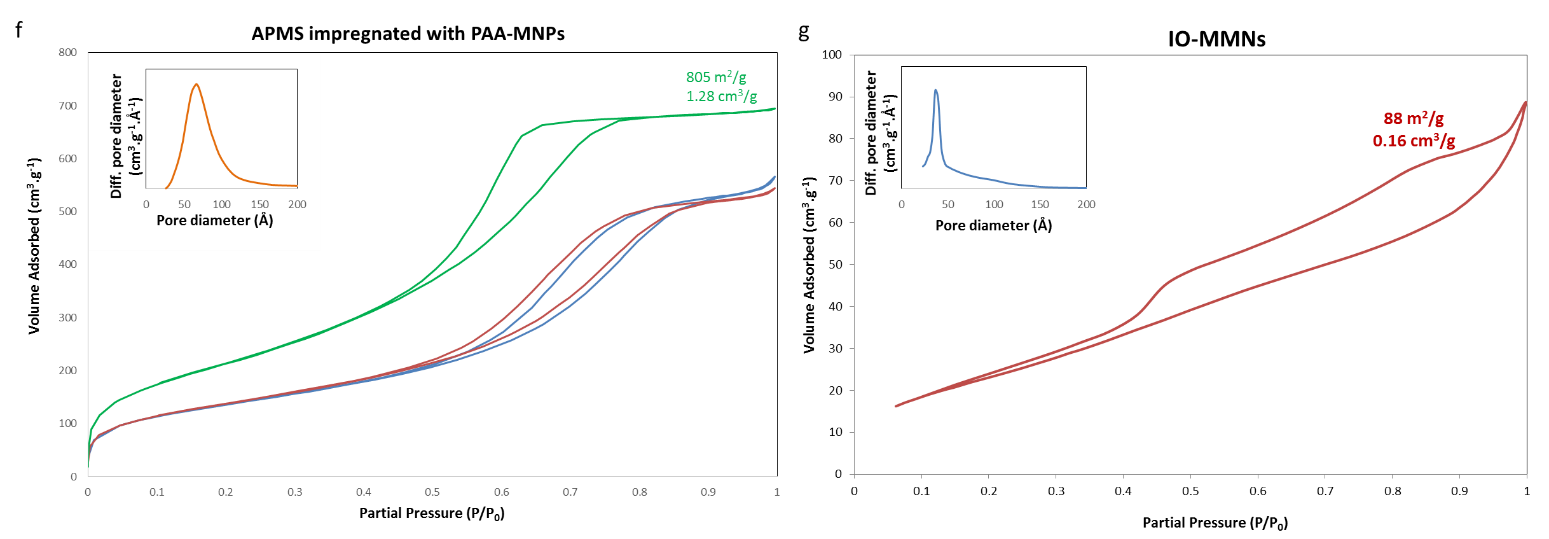
**

**h**

**Figure S2.** TEM images of (a,b) APMSimpregnated with PAA-MNPs and (c) intermediate etched with NaOH to form (d,e) iron oxide mesoporous magnetic nanoclusters (IO-MMNs). N_2_physisorption isotherm curves of (f) APMS template and its impregnation with PAA-MNPs; (g) IO-MMNs with surface area of 88 m^2^/g and pore-size volume of 0.16 cm^3^/g (inset: respective pore diameter plot). (h) DLS and zeta potential measurements of IO-MMNs dispersed in water showing average hydrodynamic size (D_H_) = 300 nm and zeta potential ξ = -15.4 m.

**a**

**
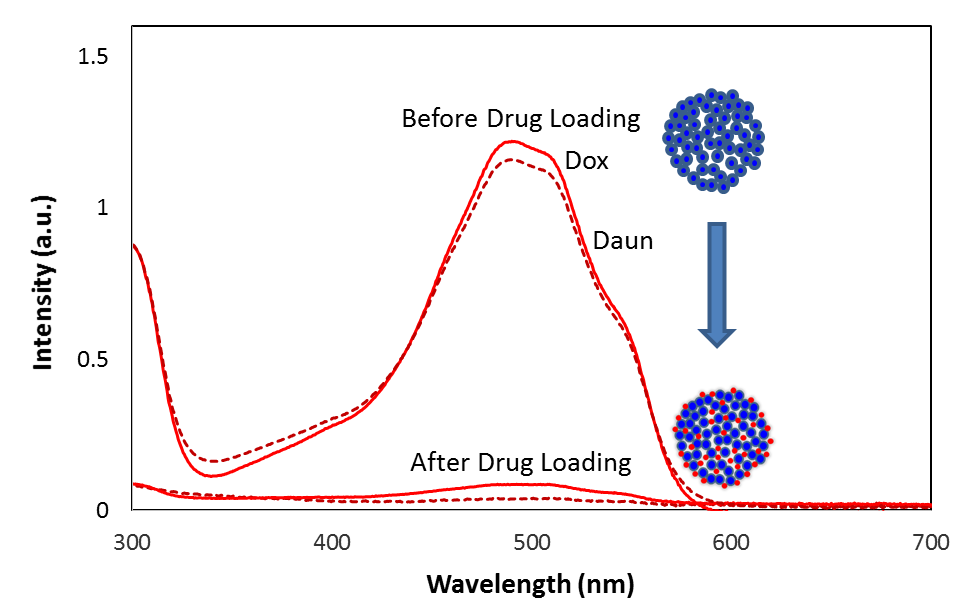

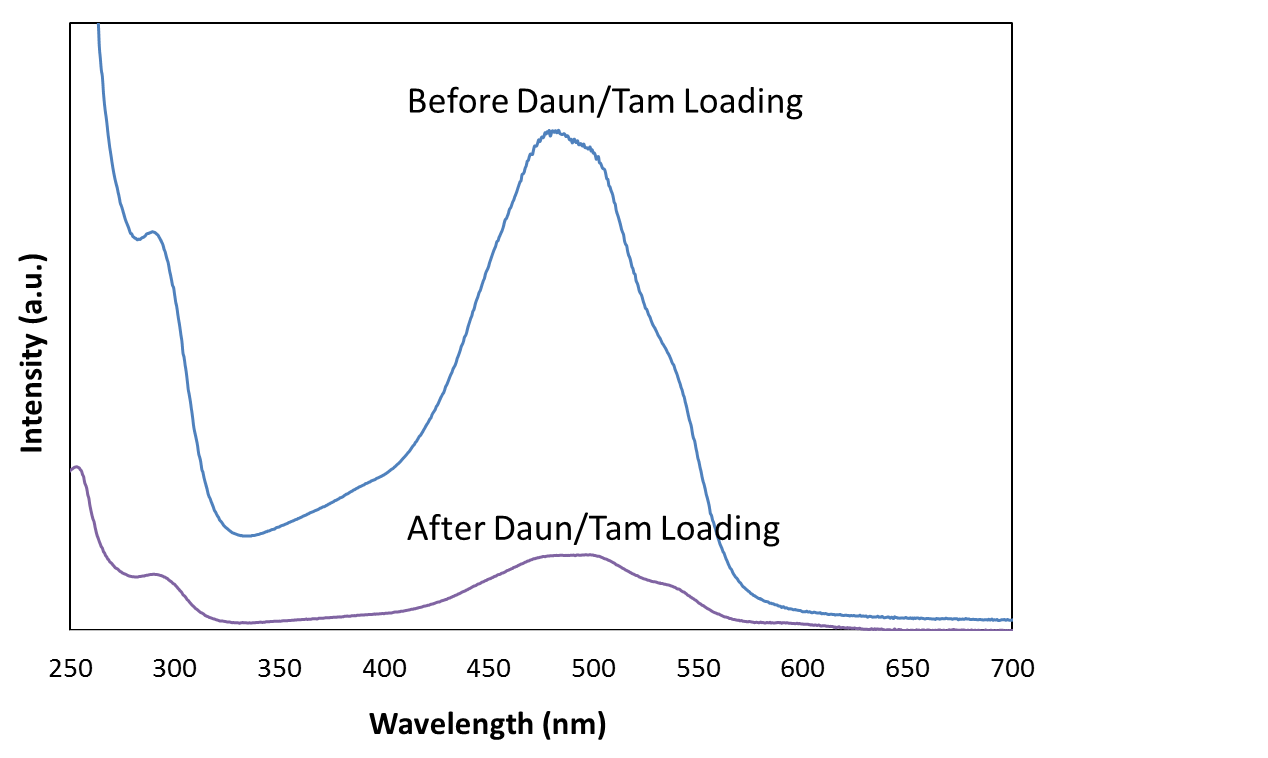
**

**b**

**
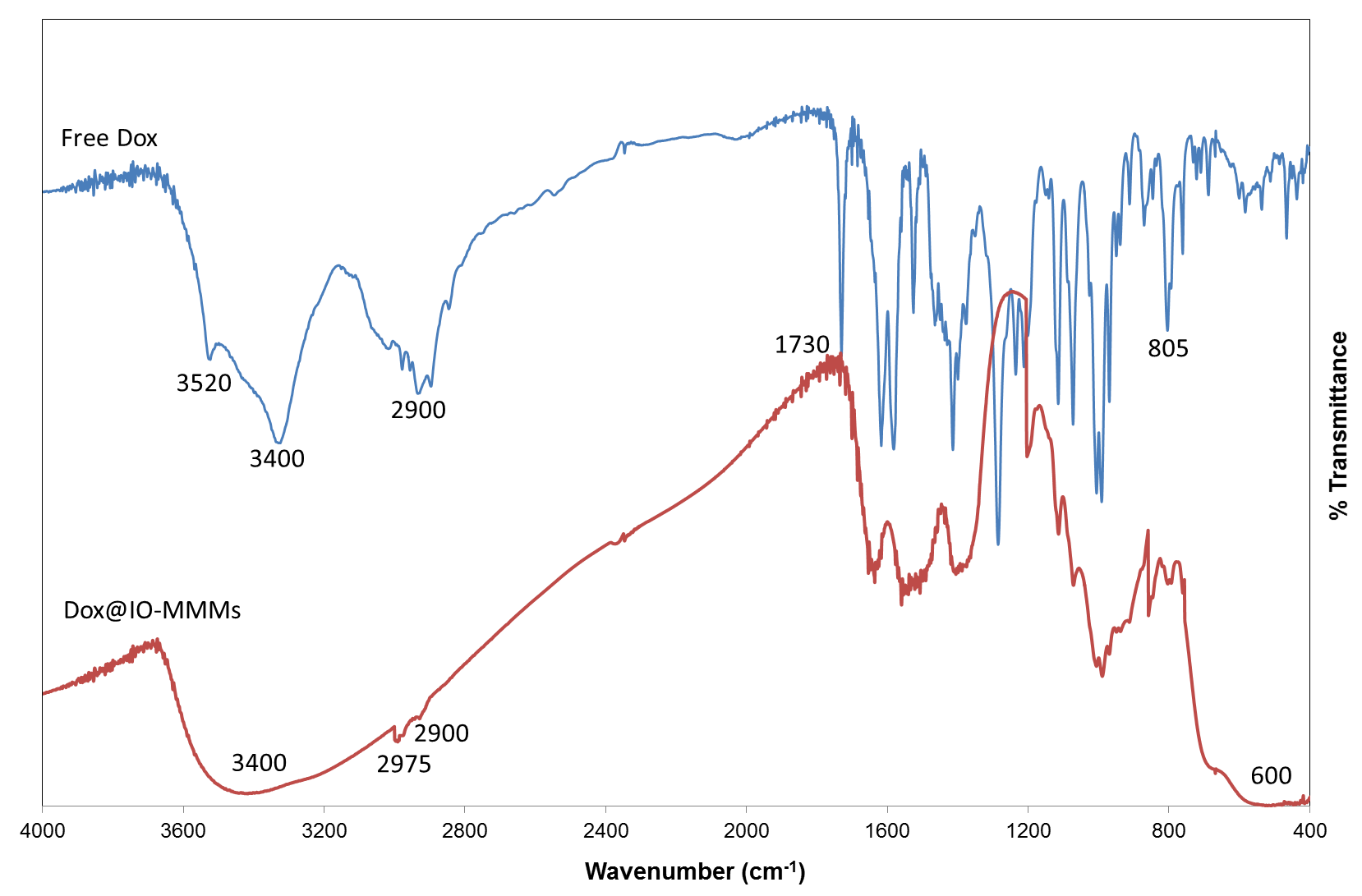
**

**Figure S3.** (a)RepresentativeUV-vis spectra of drug solutions before (initially) and afterloading clearly showing the successful incorporation of Dox, Daun, and Daun/Tam onto IO-MMMs. (b) FTIR spectra for pure free Dox (above), and Dox@IO-MMMs (below) clearly showing the peaks associated with Dox, and hence successful loading.

**a.**

**Drug@IO-MMMs**

**b.**

**Dox@IO-MMMs**

**Daun@IO-MMMs**

**Daun/Tam@IO-MMMs**

**Figure S4.** Representative hydrodynamic sizes (D_H_) and zeta potential (ξ) measurements fordrug@IO-MMMs dispersed in water. (a) Illustrative size distribution graph for drug@IO-MMMs in water with a D_H_ = 830 ± 99.95 nm showing that the particle size is not affected after drug loading. (b) Dox@IO-MMMs(ξ = - 24.9 ± 1.55 mV), Daun@IO-MMMs (ξ = - 26 ± 1.01 mV), and Daun/Tam@IO-MMMs(ξ = -7.57 ± 1.65 mV). Three independent measurements were recorded.


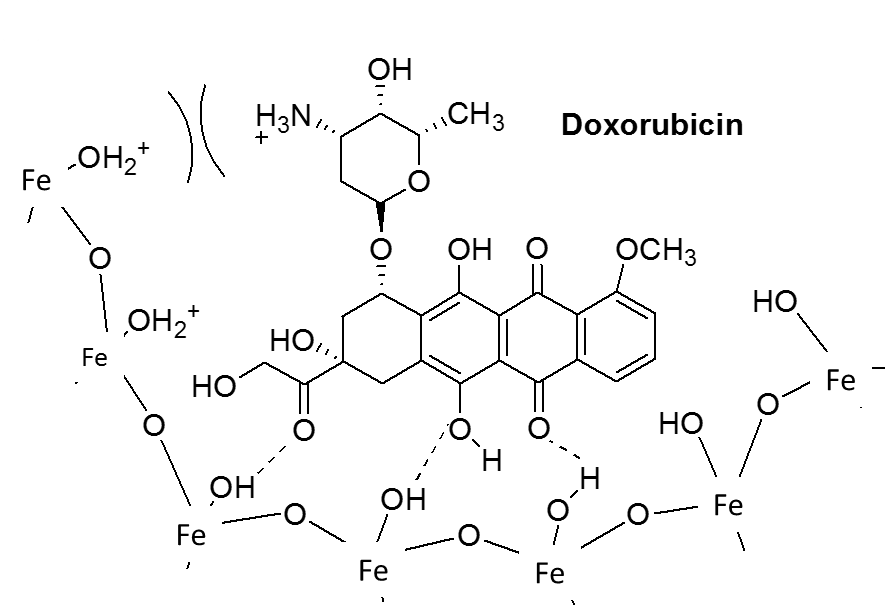


**Figure S5**. Schematic showing the mechanism of drug release from IO-MMMs due to protonation of hydroxyl groups, resulting in overall weekend bindings and dissociation from the IO-MMM surface and mesoporous channels.

**
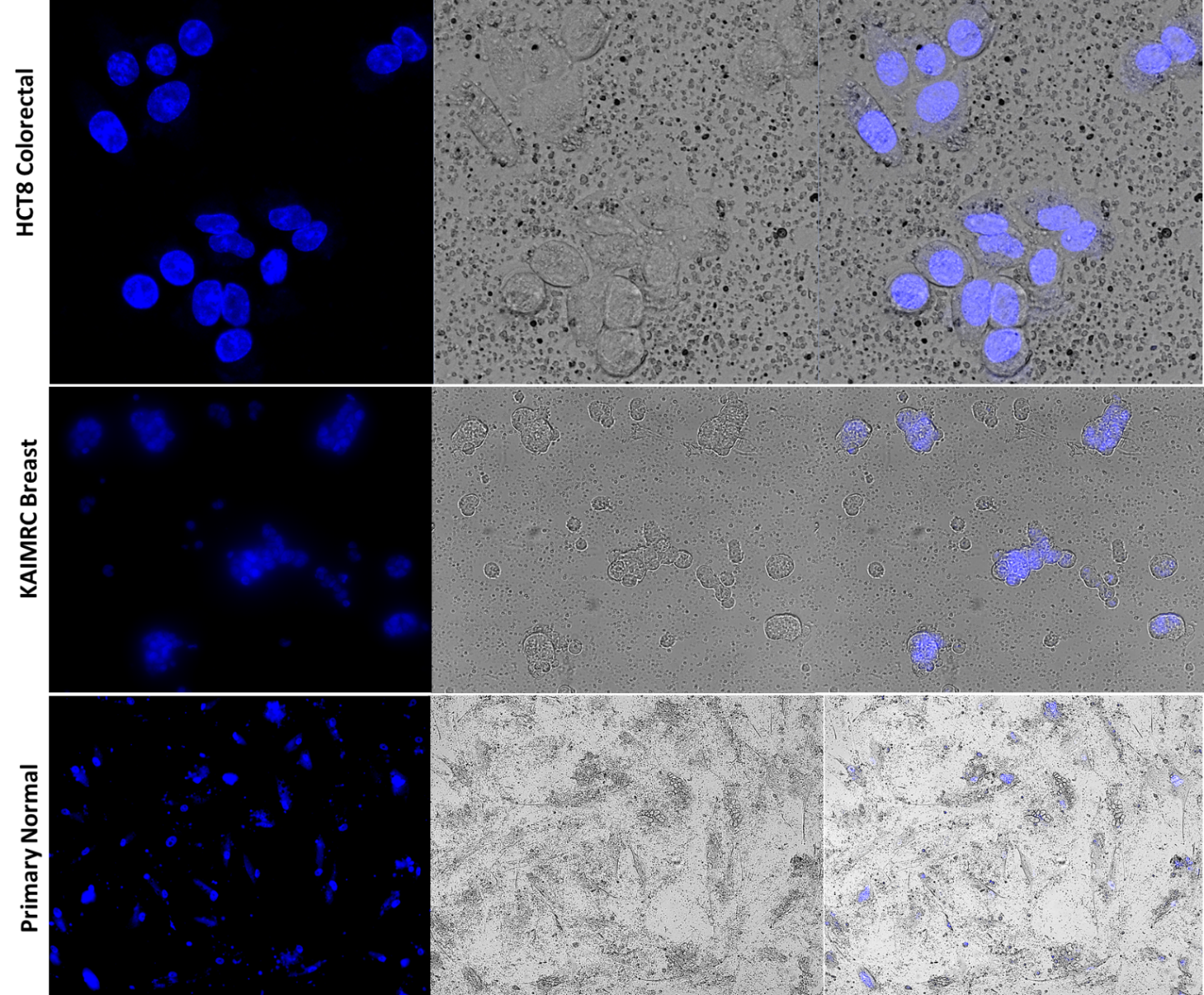
**

**Figure S6**. Confocal microscopy images of the different cells incubated with IO-MMMs(10 μg/mL particles) after 24 hrs.

(a) Hoechst channel (blue color) showing location of the nuclei; (b) transmitted light; overlay of Hoechst and transmitted light.


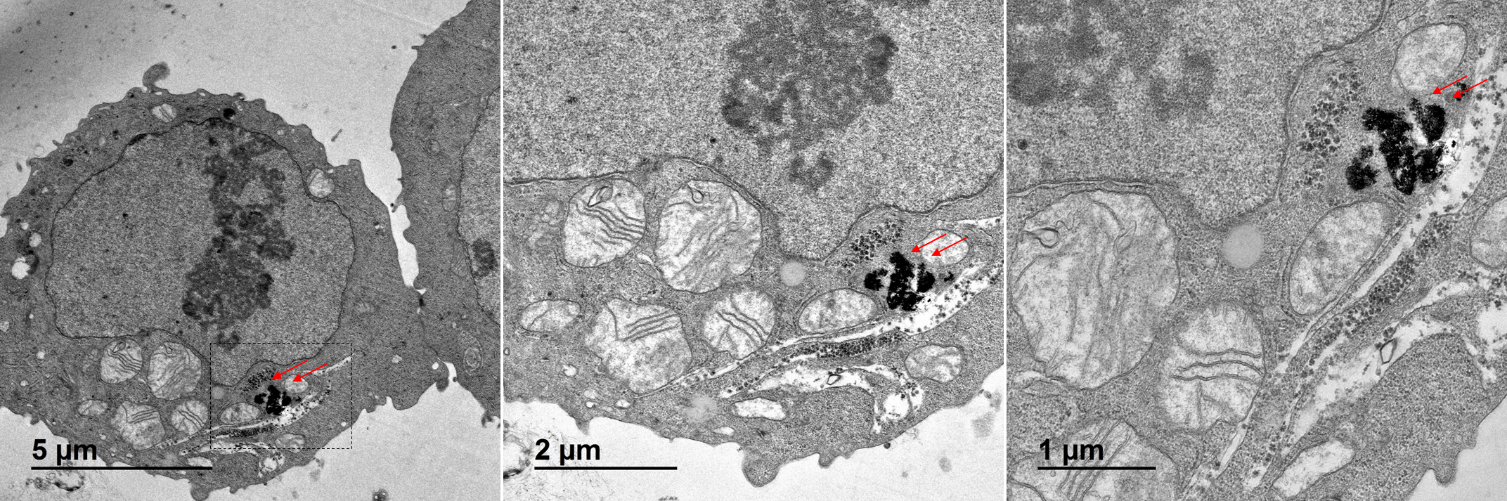


**Figure S7**. TEM images of HCT8 cells treated with IO-MMMs at different magnifications. Particles are shown in red arrowsclearly confirming the internalization inside the cytosol in compartments close to the nucleus, but not inside the nucleus.

**
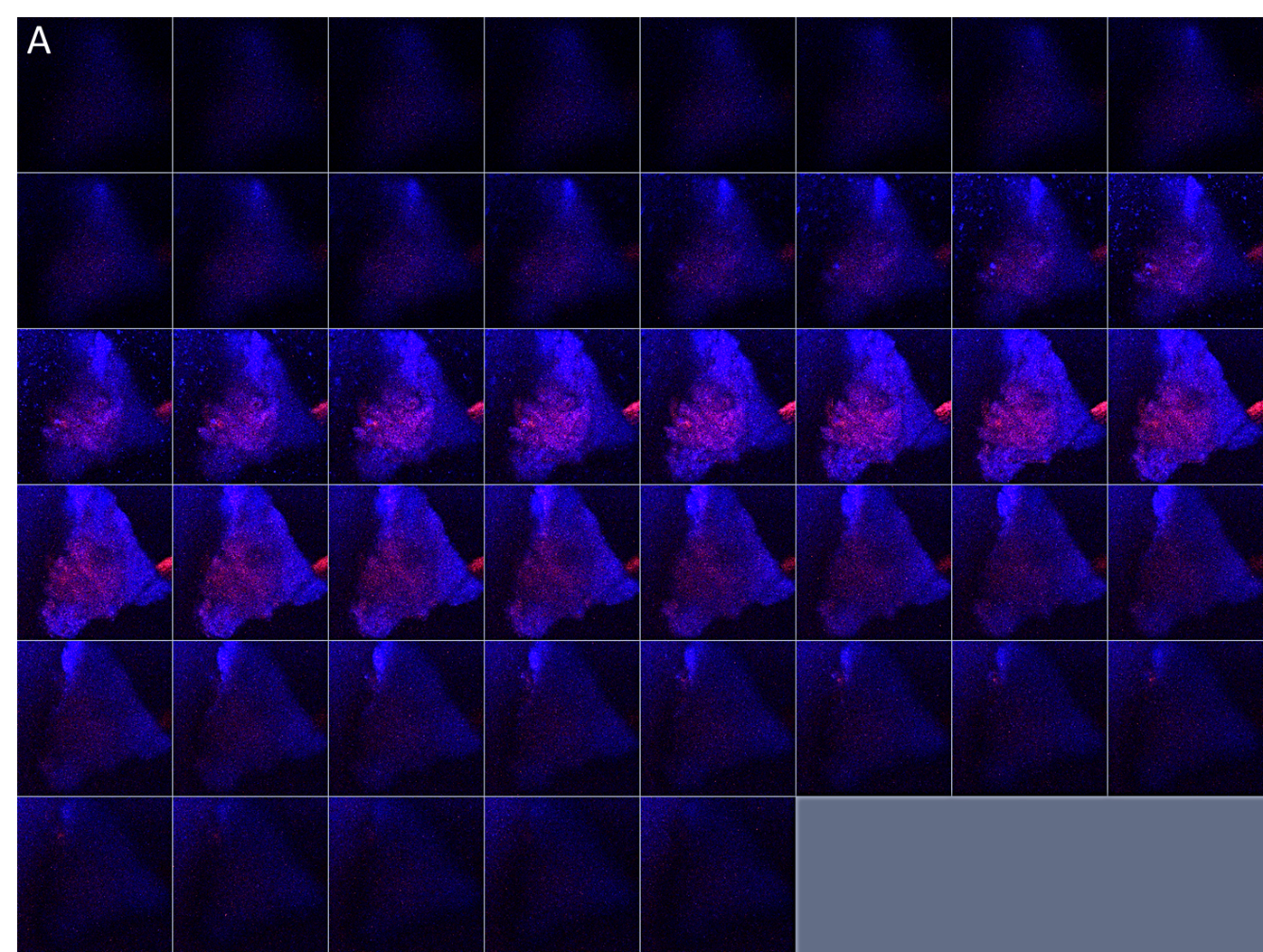
**

**
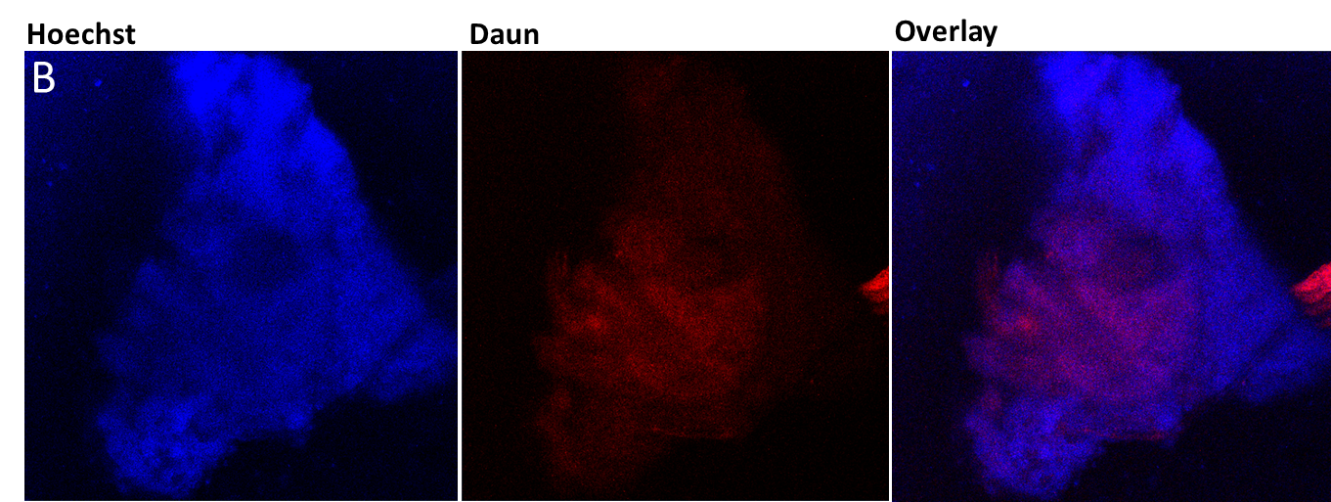
**

**Figure S8.** a)Z-stack image gallery starting from top to bottom of patient biopsy breast tumor tissue evenly cut as the tissue in Figure 9, and treated withfree Daun/Tam for 2 weeks at 37 °C and 5% CO_2_ at equivalent drug concentrations. b) Middle slice image of the z-stack images: Hoechst channel (blue); Daun channel (red); and merged overlay of both channels. Images noticeably show much lower amounts of red fluorescence (Daun), hence, limited penetration of free drugs in comparison to Daun delivered *via*Daun/Tam@IO-MMMs.
